# Neuroprotection and Mechanism of Gas-miR36-5p from *Gastrodia elata* in an Alzheimer’s Disease Model by Regulating Glycogen Synthase Kinase-3β

**DOI:** 10.3390/ijms242417295

**Published:** 2023-12-09

**Authors:** Zhongteng Lu, Jianyuan Fu, Guang Wu, Zhecheng Yang, Xiaoqi Wu, Dan Wang, Zhengying You, Zuoming Nie, Qing Sheng

**Affiliations:** College of Life Sciences and Medicine, Zhejiang Sci-Tech University, Hangzhou 310018, China

**Keywords:** Alzheimer’s disease, GSK-3β, hyperphosphorylation of tau protein, *Gastrodia elata* miRNA, Gas-miR36-5p, neuroprotection

## Abstract

Alzheimer’s disease (AD) is currently the most common neurodegenerative disease. Glycogen synthase kinase 3β (GSK-3β) is a pivotal factor in AD pathogenesis. Recent research has demonstrated that plant miRNAs exert cross-kingdom regulation on the target genes in animals. *Gastrodia elata* (*G. elata*) is a valuable traditional Chinese medicine that has significant pharmacological activity against diseases of the central nervous system (CNS). Our previous studies have indicated that *G. elata*-specific miRNA plays a cross-kingdom regulatory role for the NF-*κ*B signaling pathway in mice. In this study, further bioinformatics analysis suggested that Gas-miR36-5p targets GSK-3β. Through western blot, RT-qPCR, and assessments of T-AOC, SOD, and MDA levels, Gas-miR36-5p demonstrated its neuroprotective effects in an AD cell model. Furthermore, Gas-miR36-5p was detected in the murine brain tissues. The results of the Morris water maze test and western blot analysis provided positive evidence for reversing the learning deficits and hyperphosphorylation of Tau in AD mice, elucidating significant neuroprotective effects in an AD model following *G. elata* RNA administration. Our research emphasizes Gas-miR36-5p as a novel *G. elata*-specific miRNA with neuroprotective properties in Alzheimer’s disease by targeting GSK-3β. Consequently, our findings provide valuable insights into the cross-kingdom regulatory mechanisms underlying *G. elata*-specific miRNA, presenting a novel perspective for the treatment of Alzheimer’s disease.

## 1. Introduction

Alzheimer’s disease (AD) has become one of the most prevalent neurodegenerative disorders [1], with over 50 million individuals affected worldwide. Given the escalating global aging population, it is projected that the number of AD patients will reach 131.5 million by 2050, posing a significant latent threat to human well-being [2,3]. The amyloid hypothesis and the Tau hyperphosphorylation hypothesis are currently the two most widely accepted theories regarding the formation of Alzheimer’s disease (AD), serving as key indicators in the histopathological assessment of this disease. In this context, insoluble β-Amyloid (Aβ) aggregates give rise to amyloid plaques that accumulate in the brain, while Tau protein undergoes multisite hyperphosphorylation, leading to the formation of neuronal fiber tangles (NFTs) [4,5]. The oligomerization of hyperphosphorylated Tau subsequently results in neuronal skeleton breakage and toxicity [6], ultimately causing neurological dysfunction, neuronal cell death, and cerebral neurodegenerative lesions associated with AD [7]. Tanaka et al. [8] emphasized the critical role of translational research from in vitro to in vivo models in understanding neuro-psychiatric disorders, providing insights into potential mechanisms. Aβ accumulation and Tau hyperphosphorylation in AD further impair synapses and neural circuits [9]. Furthermore, Aβ and Tau play a crucial role in modulating the excitatory and inhibitory mechanisms of neural circuits, with an increase in excitatory time constants correlating with higher Tau deposits and an increase in inhibitory time constants notably associated with higher Aβ deposits [10,11]. Therapeutic interventions enhancing neural circuit activity show promise in ameliorating AD’s pathogenic mechanisms. Consequently, inhibiting Tau hyperphosphorylation or Aβ accumulation represents an effective preventive or therapeutic approach for AD [5,12,13].

The protein kinases directly involved in the phosphorylation of Tau protein can be broadly classified into two types based on their primary serine-directed activity. Among them, glycogen synthase kinase-3 (GSK-3), cyclin-dependent protein kinase 5 (CDK5), and mitogen-activated protein kinase (MAPK) are particularly relevant to the pathogenic mechanisms of AD [14,15,16,17]. GSK-3β, known for its crucial role in AD, can phosphorylate Tau protein at multiple sites when activated. It is preferentially co-localized with NFTs and promotes the formation of paired helical filaments (PHFs) in the brains of AD patients [18]. Therefore, research on neural substrates in AD that directly phosphorylate Tau protein holds significant importance.

**Figure 1:**
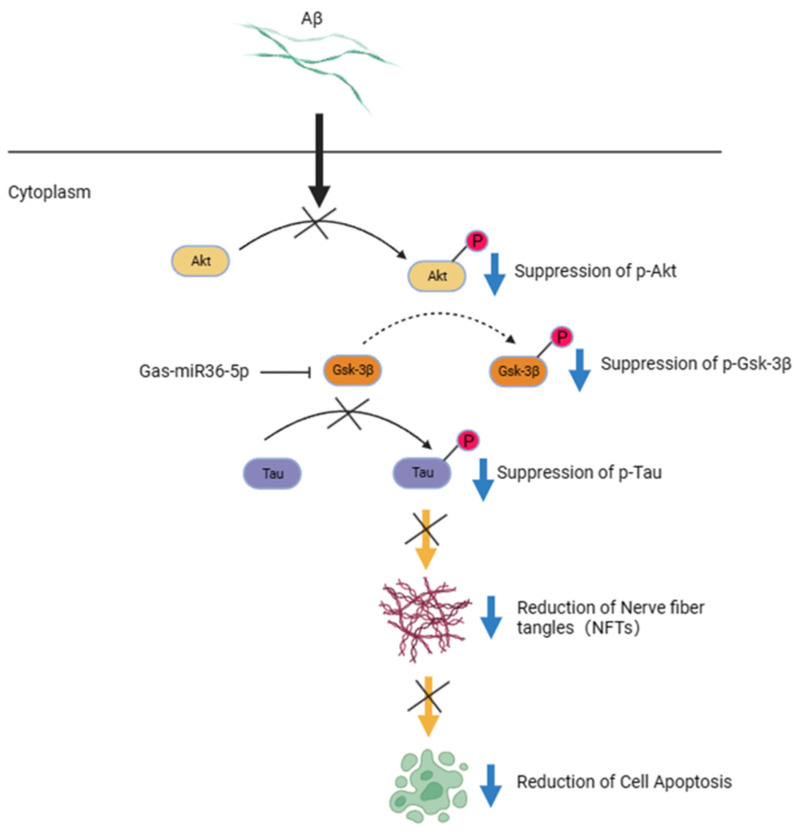
Potential mechanism of Gas-miR36-5p’s effect on nerve injury in Alzheimer’s disease (AD) cells. Amyloid β (Aβ) can downregulate Protein Kinase B (Akt) activation by inhibiting Phosphoinositide 3-kinase (PI3K) in cells. P-Akt possesses Glycogen Synthase Kinase 3β (GSK-3β) kinase activity, and the reduction in p-Akt increases the accumulation of GSK-3β kinase (active forms), resulting in Tau hyperphosphorylation and exacerbating pathological phenomena associated with AD. Gas-miR36-5p may confer neuroprotective effects through the downregulation of GSK-3β. This figure was created from BioRender.com (https://app.biorender.com/).

GSK-3β, a highly conserved serine/threonine kinase, is an isoform of the glycogen synthase kinase 3 family (GSK3s) [19]. GSK-3β is expressed in multiple tissues, with particularly abundant expression in the brain. Some studies indicate that the activity of GSK-3β in the brains of individuals with mild cognitive impairment (MCI) and AD is significantly elevated compared to healthy individuals [20]. In 1993, Koichi Ishiguro et al. [21] first demonstrated that GSK-3β possesses Tau kinase activity. GSK-3β targets multiple phosphorylation sites on Tau, with prominent sites at Ser396, Thr181, and Thr231 [22,23,24]. The aberrant activation of GSK-3β in AD leads to Tau protein hyperphosphorylation, preventing its binding to microtubules and resulting in neurodegeneration, ultimately contributing to the pathological outcomes of AD [25]. For example, when GSK-3β was overexpressed in mice, there was a significant increase in Tau phosphorylation levels within the brain [26,27]. Additionally, these mice also exhibited various behavioral deficits, such as dullness [28]. Liu et al. [29] demonstrated that miR-9-5p targets GSK-3β, and its overexpression alleviates oxidative stress and cell apoptosis induced by Aβ_25–35_. Furthermore, miR-23b-3p inhibited Tau hyperphosphorylation mediated by GSK-3β in APPswe cells and exerted a consistent neuroprotective effect in APP/PS1 transgenic mice [30]. MicroRNAs (MiRNAs) play a pivotal regulatory role in the nervous system through neural development, maturation, and maintenance. Increasing evidence suggests their significance as regulatory factors and therapeutic targets in AD. Modulation of their expression can enhance cognitive function in AD [31]. Thus, targeting GSK-3β may inhibit Tau hyperphosphorylation and halt the progression of Alzheimer’s disease [32,33,34] (Figure 1). By incorporating miRNA into the discussion, we can gain a more comprehensive understanding of its regulatory role in the nervous system and its intricate interplay with GSK-3β and other molecules associated with AD.

MiRNAs are a class of non-coding single-stranded RNA of about 18–22 nt with strong sequence conservation [35,36]. It functions by binding to the 3′-UTR of target gene mRNA with either incomplete or complete complementarity, thereby inhibiting or reducing translation into protein. This regulation occurs at both transcriptional and post-transcriptional levels [37]. Increasing evidence suggests that plant miRNAs can exert regulatory effects on animals through cross-kingdom interactions, including those derived from medicinal plants and dietary sources [38,39]. In 2012, Zhang et al. [40] discovered that miR168a from rice can downregulate the expression levels of low-density lipoprotein receptor adapter protein 1 (LDLRAP1) mRNA in human and mouse cells. In 2014, Zhang’s team identified a stable miRNA, miR2911, present in honeysuckle decoction, which targets key genes in various subtypes of influenza viruses, including H5N1 and H1N1. The experiment demonstrated that miR2911 influences virus replication and reduces mortality rates in mice infected with H5N1 [41]. In 2015, Chin et al. [42] discovered that plant-derived miR159 was present in human serum and showed a negative correlation with the progression of breast cancer. Through in vivo and in vitro experiments, they provided the first evidence that plant-derived miR159 inhibits breast cancer cell proliferation through cross-kingdom regulation. Recently, numerous experiments have demonstrated the significant potential of plant-derived miRNAs in regulating mammalian gene expression and even treating diseases. Therefore, we are particularly interested in exploring plant-derived miRNAs, especially those from traditional Chinese herbal medicine.

*Gastrodia elata Blume* (*G. elata*) is a valuable Chinese medicine with a history spanning nearly two thousand years. *G. elata* exhibits significant pharmacological efficacy in the treatment of central nervous system diseases, and extensive research has been conducted on its major components, including gastrodin, polysaccharides, vanillin, and other active compounds [43,44]. However, the miRNAs of *G. elata* have been poorly investigated. Our previous studies have confirmed that the specific miRNAs, miRNA01 and miRNA02, from *G. elata* exhibit anti-inflammatory effects by modulating the NF-*κ*B pathway. Building upon this research [45], we hypothesize that miRNAs from *G. elata* may play corresponding roles in central nervous system disorders. Our group early on screened out 38 novel *G. elata*-specific miRNAs through sequencing technology and ultimately identified a candidate *G. elata*-specific miRNA called Gas-miR36-5p that targets GSK-3β. Based on previous work, we investigated the impact of Gas-miR36-5p targeting the GSK-3β signaling pathway in vitro and in vivo. Our aim was to demonstrate that *G. elata*-specific miRNA can regulate GSK-3β, leading to the inhibition of Tau protein hyperphosphorylation and ultimately providing neuroprotection against AD.

This study will help to reveal the molecular-level medicinal mechanism of *G. elata*-specific miRNA on CNS diseases and establish a theoretical basis for the use of *Gastrodia elata*-derived miRNA as a pharmacodynamic substance.

## 2. Results

### 2.1. Gas-miR-36-5p Regulates GSK-3β Expression by Directly Binding to the 3′-Untranslated Regions

**Figure 2:**
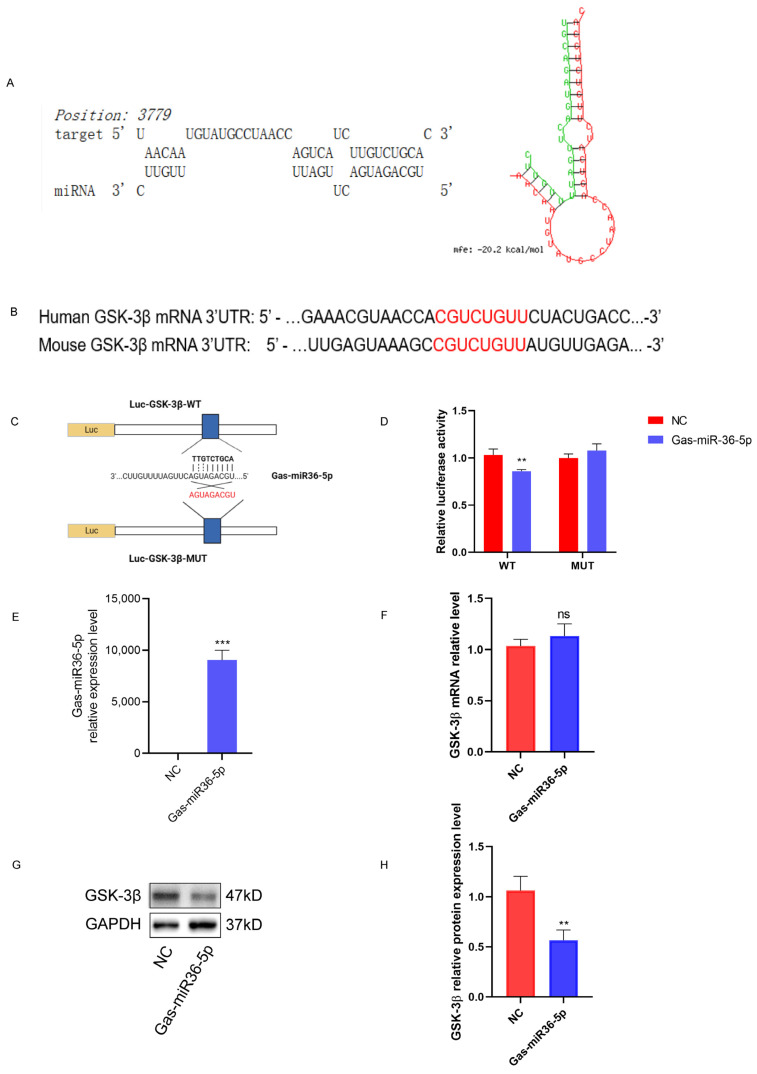
Verification of Gas-miR36-5p targeting GSK3β. (**A**) Bioinformatics software predicts the potential target and the combined site and structure. (**B**) Comparison of homology between humans and mice. The red font indicates the presence of the same target site in both humans and mice. (**C**) According to the binding sites given by the software, the connecting sequence of wild and mutant groups was designed. (**D**) A dual-luciferase reporter gene assay determined the binding of the target. (**E**,**F**) RT-qPCR was used to detect the transfection efficiency of Gas-miR36-5p and the expression level of GSK-3β mRNA in human neuroblastoma SH-SY5Y (SH-SY5Y) cells. (**G**,**H**) A western blot experiment was used to detect the protein expression level of GSK-3β after transfection of Gas-miR36-5p. *ns*, no significance; ** *p* < 0.01; *** *p* < 0.001.

Based on the results of miRmap, RNA22v2, and RNAhybrid prediction software (See these website links in Section 4.3), we identified a candidate miRNA from the previously identified *G. elata*-specific miRNAs that has the potential to target GSK-3β. Gas-miR36-5p exhibited a reliable target according to the prediction software analysis, displaying a relatively low binding energy with the GSK-3β mRNA 3′-UTR binding site. The possible binding modes of Gas-miR36-5p and GSK-3β mRNA were predicted using RNAhybrid prediction software (Figure 2A). Additionally, sequence alignment revealed that the binding site sequences of Gas-miR36-5p on human and mouse GSK-3β mRNA 3′-UTR were identical (Figure 2B), indicating interspecific conservation. This finding establishes a solid foundation for the subsequent in vivo and in vitro experiments.

The result of the dual-luciferase reporter gene assay demonstrated that co-transfection with Gas-miR36-5p mimics and p-mirGLO-GSK-3β-3′ UTR-WT cells led to a significant reduction in dual luciferase activities compared to the MUT group. This finding indicates that Gas-miR36-5p specifically targets and downregulates the expression of the p-mirGLO-GSK-3β-3′ UTR-WT firefly luciferase reporter gene, thereby confirming its targeting ability towards GSK-3β mRNA (Figure 2C,D).

Initially, the RT-qPCR experiment confirmed that Gas-miR36-5p mimics could be efficiently transfected into the AD cell model SH-SY5Y cells using liposomes (Figure 2E). Then the following results showed that transfection of Gas-miR36-5p mimics led to a certain downregulation of GSK-3β at the protein level in SH-SY5Y cells, although no significant difference was observed at the transcriptional level (Figure 2F–H).

To summarize, our results indicated that GSK-3β is a target of Gas-miR36-5p. Although it does not significantly affect the expression of GSK-3β mRNA, it may downregulate the protein level of GSK-3β by inhibiting mRNA translation at the post-transcriptional level. Further investigations on Gas-miR36-5p will be conducted in future studies.

### 2.2. Oxidative Stress Indexes Show That Gas-miR36-5p Can Exert Neuroprotection in SH-SY5Y

**Figure 3:**
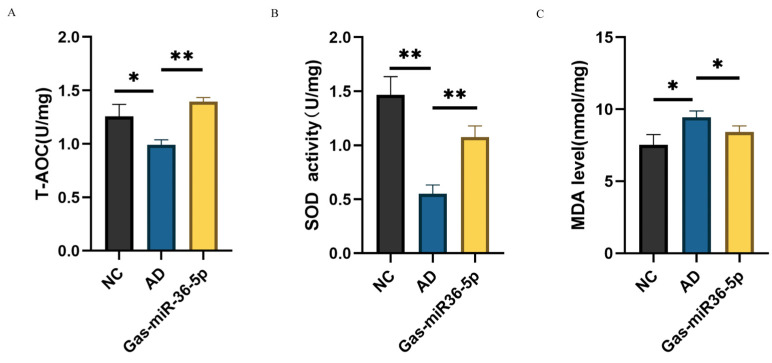
Effect of Gas-miR36-5p on the oxidative stress index of the AD cell model. Results of Total Antioxidant Capacity (T-AOC) (**A**), Superoxide Dismutase (SOD) (**B**), and Malondialdehyde (MDA) (**C**) after transfection of Gas-miR36-5p in an Aβ-induced AD cell model. * *p* < 0.05; ** *p* < 0.01.

Compared to the NC group, we assessed the Total Antioxidant Capacity, Superoxide Dismutase, and Malondialdehyde (T-AOC/SOD/MDA) indicators in the AD cell model and observed a decrease in T-AOC and SOD levels along with an increase in MDA level after induction with Aβ_25–35_. These findings indicate that Aβ_25–35_ has a pronounced impact on SH-SY5Y cells post-induction, thereby validating the success of our modeling approach. Subsequently, following transfection with Gas-miR36-5p, we conducted a reassessment of the T-AOC/SOD/MDA levels and observed a reversal in the reductions in T-AOC and SOD induced by modeling (Figure 3A,B). Additionally, there was also a reversal in the elevation of MDA levels (Figure 3C), indicating that Gas-miR36-5p effectively counteracts the neurotoxicity caused by Aβ_25–35_ treatment in AD cell models. These findings suggest that Gas-miR36-5p exhibits neuroprotective effects, at least based on these cellular indicators.

### 2.3. Gas-miR36-5p Suppresses Tau Phosphorylation in an AD Cell Model by Reducing the Activation of GSK-3β

**Figure 4:**
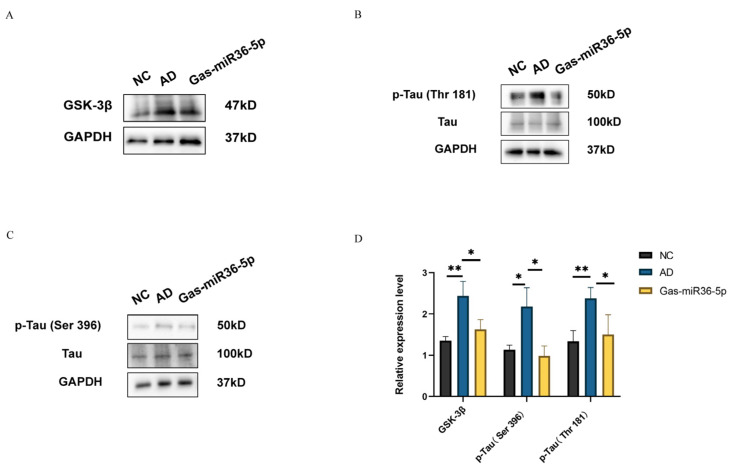
The expression level of AD-related protein in the SH-SY5Y AD cell model. A western blot was used to detect the expression levels of GSK-3β (**A**), p-Tau (Thr181) (**B**), and p-Tau (Ser396) (**C**) in the AD cell model after transfection with Gas-miR36-5p. (**D**) Western Blot analysis results.* *p* < 0.05; ** *p* < 0.01.

We assessed the protein expression level of GSK-3β and the activation status of two phosphorylation sites (Thr181 and Ser396) on Tau protein following a 24-h treatment with Aβ_25–35_. Compared to the NC group, both the protein expression level of GSK-3β and the two phosphorylated statuses of Tau protein at these two sites were observed to be significantly upregulated upon exposure to Aβ_25–35_. Furthermore, following transfection, we observed a decrease in GSK-3β activation (Figure 4A) and a reduction in the ratios of p-Tau (Thr181)/Tau and p-Tau (Ser396)/Tau in the transfected Gas-miR36-5p mimics group compared to NC mimics (Figure 4B,C). These findings indicate a decreased phosphorylation level of Tau at these two phosphorylation sites. The above results demonstrate that Gas-miR36-5p exerts its inhibitory effect on the GSK-3β pathway by directly targeting GSK-3β. As a kinase of Tau protein, GSK-3β plays a role in suppressing Tau phosphorylation. In summary, the results confirm that Gas-miR36-5p effectively mitigates the detrimental effects induced by Aβ_25–35_ in vitro, thereby establishing its protective potential on SH-SY5Y cells. Moreover, this study lays the groundwork for future investigations into the therapeutic impact of Gas-miR36-5p on animal models of Alzheimer’s disease at an in vivo level.

### 2.4. Effect of Total RNA Extract from G. elata on Behavior in an AD Mouse Model

**Figure 5:**
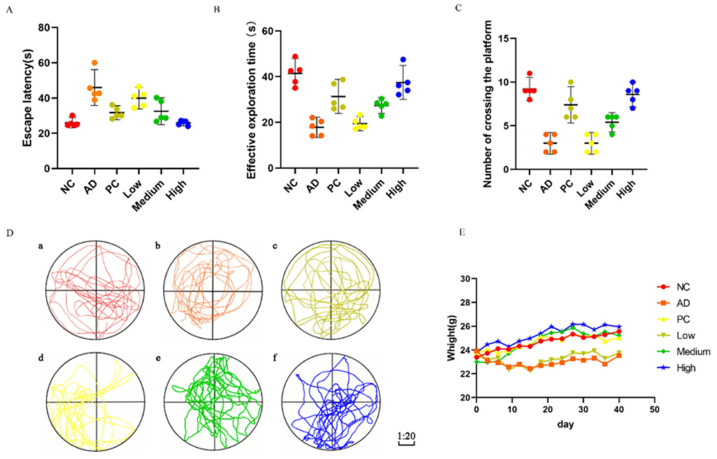
The test behavior of differentially treated mice. The escape latency (**A**), effective exploration time (**B**), and platform crossing times (**C**) of mice were detected for the different groups. (**D**) Trace diagrams of the space exploration of mice during the experiment: (**a**): Negative control (NC) group, exploration distance (ED): 237 cm; (**b**): Alzheimer’s disease (AD) group, ED: 74 cm; (**c**): Phencyclidine (PC) group, ED: 233 cm; (**d**): low-dose (Low) group, ED: 108 cm; (**e**): medium-dose (Medium) group, ED: 141 cm; (**f**): high-dose (High) group, ED: 173 cm. Upper left: the first quadrant; upper right: the second quadrant; lower left: the third quadrant; and lower right: the fourth quadrant. The fourth quadrant is the target quadrant. (**E**) With different treatments, the weight changes in mice were recorded.

The mice in each group demonstrated normal vision and motor abilities, as evidenced by their accurate localization and swimming towards the platform in the visual platform experiment. This provided a solid foundation for subsequent spatial navigation and exploration experiments. During the spatial navigation experiment, the Alzheimer’s disease (AD) group mice exhibited significantly prolonged latency in locating and swimming toward the platform compared to the negative control (NC) group, suggesting impaired formation of platform memory in AD mice, possibly attributable to inherent memory deficits resulting from AD modeling. The success of the AD mouse model was further confirmed by these findings. The Phencyclidine (PC) group, treated with the AD-positive drug donepezil hydrochloride, exhibited a significantly shorter escape latency than the AD group, indicating the substantial improvement in learning and memory deficits observed in the AD mouse model. The escape latency gradually decreased with increasing intragastric concentrations of *G. elata* total RNA in the low-dose, medium-dose, and high-dose (Low/Medium/High) *G. elata* total RNA groups. In the high-dose group, the escape latency was nearly equivalent to that of the NC group, indicating a significant enhancement in learning and memory abilities in AD mice following treatment with *G. elata* total RNA (Figure 5A).

During the spatial probe test, the AD mice exhibited significantly shorter probe times than the NC group, indicating impaired memory function. The PC group, treated with donepezil hydrochloride, demonstrated slightly longer probe times than the NC group, suggesting partial improvement in cognitive performance. In contrast, the Low/Medium/High *G. elata* total RNA groups showed extended probe times, highlighting RNA’s potential to enhance learning and memory abilities in AD mice and potentially alleviating cognitive impairments (Figure 5B). The number of platform crossings observed in each group followed similar trends as the probe times (Figure 5C).

Subsequently, we generated trace diagrams illustrating the swimming paths of mice in the Morris maze (Figure 5D). These diagrams depicted the swimming traces of mice from different groups, including NC, AD, PC, Low, Medium, and High, respectively. The target quadrant was represented by the fourth quadrant, located at the bottom right corner. Notably, within this quadrant, exploration traces of mice belonging to the High group as well as those in the NC and PC groups exhibited higher intensity compared to other quadrants. In contrast, the exploration traces of mice in the AD and Low groups were sparsely observed in the fourth quadrant. A comparison of effective exploration distance (ED) among different groups revealed that the NC and PC groups exhibited significantly greater exploration distances in the target quadrant compared to those of the other groups. ED increased with higher total RNA concentrations in the Low/Medium/High *G. elata* total RNA groups, indicating that these groups demonstrated relatively stable memory of the platform’s position compared to the AD group.

Additionally, following three days of modeling, we observed a significant reduction in body weight within the AD and Low groups compared to the other groups starting from the ninth day onward. No notable differences were detected among the NC, PC, Medium, and High groups (Figure 5E). This consistent trend persisted until the 40th day, indicating that *G. elata* total RNA provided protection against D-galactose and AlCl_3_-induced injuries in mice, thereby confirming successful AD mouse modeling to some extent.

In summary, based on the outcomes of an animal behavioral experiment, both the high-dose *G. elata* total RNA group and the donepezil hydrochloride group exhibited significant improvement in cognitive impairments induced by the combination model of D-galactose and AlCl_3_ in AD mice.

### 2.5. Regulation of G. elata-specific Gas-miR36-5p on the GSK-3β Signaling Pathway in an AD Mouse Model

**Figure 6:**
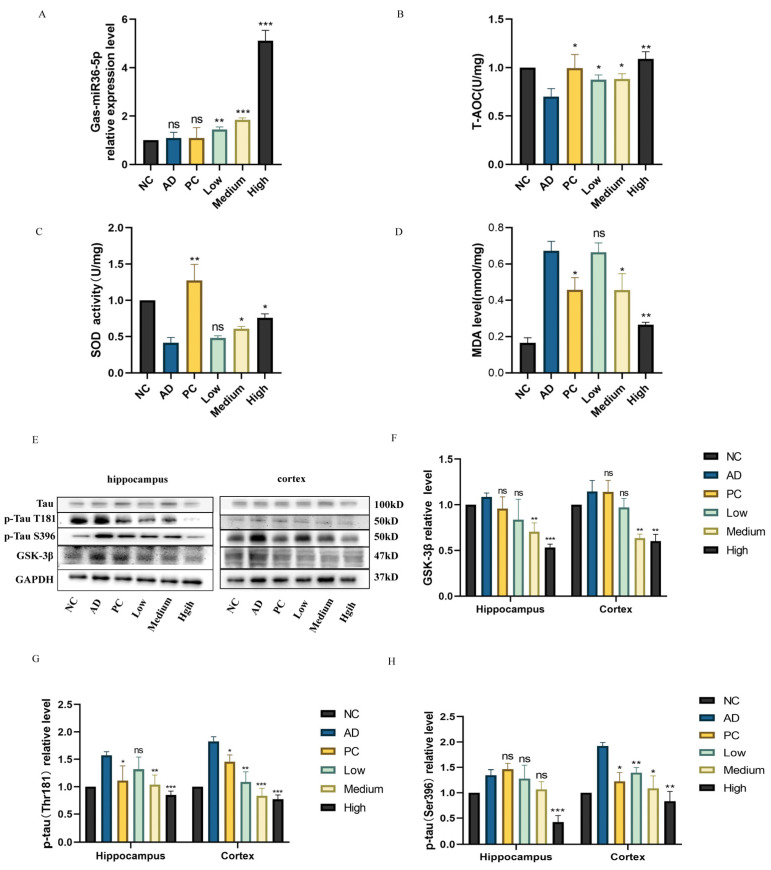
Molecular mechanism in AD model mice. (**A**) Detection of Gas-miR36-5p in the brains of different gastric perfusion groups. Detection of oxidative stress indexes related to T-AOC (**B**), SOD (**C**), and MDA (**D**) in mouse brain tissues. Expression of GSK-3β protein level and Tau (Thr181 and Ser396 site) phosphorylation level in mouse brain tissue (**E**) and analysis of western blot results (**F**–**H**). *ns*, no significance; * *p* < 0.05; ** *p* < 0.01; *** *p* < 0.001, compared to the AD model group, *n* = 6.

Initially, we predicted a potential interaction between Gas-miR36-5p and the 3′UTR region of mouse GSK-3β, implying the potential role of Gas-miR36-5p in modulating GSK-3β expression in mice. Subsequently, through RT-qPCR analysis, the presence of Gas-miR36-5p in mouse brains was detected in all groups that received *G. elata* total RNA, with the highest levels observed in the high-dose group, indicating dose-dependent delivery of Gas-miR36-5p into mouse brain tissues (Figure 6A). The assessment of oxidative stress markers (T-AOC, SOD, and MDA) showed that AD model mice had decreased T-AOC and SOD levels, while MDA levels increased. Treatment with *G. elata* total RNA and donepezil hydrochloride reversed these changes by restoring T-AOC and SOD levels and reducing MDA levels (Figure 6B–D).

The expression of the GSK-3β protein in mouse brain tissues was subsequently assessed using western blot analysis. In the AD model group, GSK-3β expression noticeably increased, consistent with AD symptoms. Donepezil hydrochloride, just as a positive control drug for AD in the PC group, can increase brain acetylcholine concentration without affecting GSK-3β. The protein levels of GSK-3β decreased with increasing dosages of *G. elata* total RNA (low, medium, and high), especially in the medium-dose and high-dose groups, suggesting a potential regulatory role of Gas-miR36-5p on GSK-3β in vivo. Given that GSK-3β possesses Tau protein kinase activity, its downregulation may contribute to the regulation of Tau hyperphosphorylation in AD.

We observed a significant increase in phosphorylation of the Tau protein at Thr181 and Ser396 in the AD model group compared to the NC group. In the *G. elata* total RNA groups, there was a significant downregulation of phosphorylation at these two sites, with an increase in doses (low, medium, and high) in the mouse brain cortex. Similarly, phosphorylation of the Tau protein at Thr181 and Ser396 was significantly downregulated in the hippocampus of the mouse brain in the medium-dose and high-dose groups of *G. elata* total RNA (Figure 6E–H), supporting our in vitro findings. The high-dose group exhibited the most pronounced effects in both the hippocampus and the cortex, confirming our in vitro findings.

The observed behavioral improvements in AD mice corresponded to the molecular-level findings, with the high-dose *G. elata* total RNA group showing the most favorable outcomes in memory and behavior. These results confirm the neuroprotective potential of *G. elata* RNA and suggest a role for Gas-miR36-5p in regulating AD in mice, paving the way for further investigations into the therapeutic potential of *G. elata* miRNA in combating diseases.

## 3. Discussion

MiRNAs are involved in the regulation of post-transcriptional gene expression in animals and plants. In recent years, the cross-kingdom regulation of miRNA has become a new frontier field. *Gastrodia elata*, as a traditional Chinese medicine, has pharmacological effects on the central nervous system (CNS). So, what kind of role do miRNAs from *G. elata* have as a new medicine substance? In our previous study, we conducted a comprehensive screening and successfully identified 38 *G. elata*-specific miRNAs. Furthermore, we demonstrated that Gas-miR01 and Gas-miR02 exert a positive regulatory effect on inflammation by specifically targeting A20 within the NF-κB pathway [45]. The present study demonstrates that Gas-miR36-5p can effectively target GSK-3β, a pivotal protein implicated in the pathogenesis of AD, as revealed by bioinformatics analysis of *G. elata* miRNAs. Consequently, we postulate that Gas-miR36-5p may exert a crucial role in neuroprotection against AD. Currently, the regulation of AD primarily involves endogenous miRNAs [46,47], with limited research on the exogenous miRNAs involved in cross-kingdom regulation of AD in addition to our previous research. In recent years, there has been an increasing focus on the role of miRNAs in disease [48,49,50]. Additionally, apart from their significant roles in plant growth, the study of plant miRNAs in cross-kingdom regulation has also made important strides [51,52,53]. At present, it has been observed that a greater number of plant miRNAs can persist within animal organisms and exert biological functions, potentially even exhibiting positive effects in certain diseases [54,55]. The miRNAs derived from the Happy Tree may regulate the signal pathway of tumor development [56]. Similarly, there is a negative correlation between the abundance of miR-159 in serum and the incidence of breast cancer [42]. Notably, honeysuckle-derived miR2911 exhibits the function of cross-kingdom virus replication inhibition in animals [41,57].

In this study, we focused on GSK-3β, a pivotal protein in AD pathology, to investigate the protective mechanisms of *G. elata*-specific Gas-miR36-5p in both in vitro and in vivo AD models. In initial in vitro experiments, Gas-miR36-5p effectively reduced GSK-3β protein expression at the cellular level by targeting the 3′-UTR of GSK-3β mRNA. Consequently, this led to a decrease in Tau protein over-phosphorylation at critical sites (Thr181 and Ser396) in the AD cell model. Given that excessive phosphorylation of Tau is a critical factor in AD pathogenesis, these findings robustly support the neuroprotective role of Gas-miR36-5p in the AD cell model. Importantly, these results not only confirm the neuroprotective effect of Gas-miR36-5p in the AD cell model but also establish a foundation for its impact in vivo in an AD mouse model. Additionally, intragastric administration of *G. elata* RNA in the AD mouse model confirmed improvements in learning and memory abilities at a behavioral level. The molecular-level observations confirmed the neuroprotective role of *G. elata* RNA and suggested that Gas-miR36-5p may regulate GSK-3β in an AD mouse model, indicating the potential for further exploration of cross-kingdom regulation of *G. elata* miRNA in diseases.

It is worth exploring the potential functions of plant miRNAs, especially those from traditional Chinese medicine, in animals’ bodies. Plant miRNAs have shown increasingly positive therapeutic potential in various diseases [39]. Understanding the precise roles of these plant miRNAs that can stably exist in animal bodies or considering them as novel active ingredients in traditional Chinese medicine can reveal the mechanisms behind herbal medicine’s therapeutic effects. We conducted in vivo studies by intragastric administration of *G. elata* total RNA. However, to investigate the function of Gas-miR36-5p more precisely, additional methods or specialized gastric infusions should be employed. Nevertheless, due to the limited conditions, it is very difficult to scale the preparation of Gas-miR36-5p for in vivo experiments. Thus, this study utilized *G. elata* total RNA to explore its impact in an AD mouse model. We administered a 3 mg/kg *G. elata* total RNA dosage based on a thorough literature review [40,42]. The experiment results only provide the possibility of demonstrating the effect of *G. elata* miRNA. However, further investigation will be conducted with improved preparation on the potential neuroprotective effects by avoiding non-specific results. In our research on *G. elata* miRNAs, Gas-miR36-5p, as a small molecule, may play a neuroprotective role in AD. However, the specific mechanism of action for each miRNA may be complex. Our goal is to understand the precise mechanisms and therapeutic potential of *G. elata* as a whole by studying individual miRNAs. Gas-miR36-5p, identified as a novel active ingredient in *G. elata*, provides important clues for understanding the mechanisms underlying traditional Chinese medicine.

Currently, the Aβ and Tau protein hypotheses have become the most popular and significant theories in AD pathology. Increasing evidence suggests that the abnormal accumulation of Aβ and Tau proteins may be interconnected, with intersecting points in AD pathogenesis [58]. Aβ aggregation may further exacerbate neuronal damage by activating abnormal phosphorylation of Tau protein, ultimately manifesting as clinical symptoms of AD [59]. However, both hypotheses have certain limitations. Firstly, current treatments targeting either the Aβ-related pathway or Tau protein hyperphosphorylation have not achieved the desired success, possibly due to the fact that neither the Aβ hypothesis nor the Tau hypothesis alone can fully explain the origin and development of AD. Therefore, a comprehensive understanding of AD pathogenesis may necessitate considering the interaction between Aβ and Tau proteins with other potential factors. Future research should focus more on the intersections between these hypotheses to gain a holistic understanding of the pathophysiology of AD. If drugs could target multiple points for treating AD and potentially yield better results, it aligns with TCM’s pharmacological characteristic of acting on multiple targets [60]. *G. elata*, a TCM used for neurodegenerative diseases since ancient times, shows potential in AD. Identifying its specific components is crucial to understanding the precise role of *G. elata* in AD.

So far, research on *G. elata* primarily focuses on its compound composition [61]. Given the growing interest in cross-disciplinary regulation, miRNAs, as small molecules with significant regulatory functions, have sparked enthusiasm for exploring their roles within *G. elata*. Given the complexity of active ingredients in traditional Chinese medicine, it is crucial to elucidate the specific functions of individual components. In our study, we focused on Gas-miR36-5p, a miRNA derived from *G. elata*, and demonstrated for the first time that it may exert neuroprotective effects in an AD model by targeting GSK-3β. This is a promising discovery. Based on in vitro and in vivo experiments, we provide new evidence for the theory of cross-kingdom regulation. Additionally, we establish that *G. elata* miRNA (Gas-miR36-5p) can regulate the expression of GSK-3β across different kingdoms, playing a neuroprotective role. However, it is currently in the experimental stage, and further research is required to validate this finding, considering dosage and potential side effects. In summary, our research offers a potential avenue for AD treatment and lays the foundation for understanding *G. elata*’s role in neurodegenerative diseases. MiRNA, a novel active ingredient found in *G. elata*, has significant implications for advancing our understanding of traditional Chinese medicine mechanisms.

## 4. Materials and Methods

### 4.1. Cell Culture and Transfection

The SH-SY5Y cells were cultured in Dulbecco’s modified Eagle’s medium (DMEM, Gibco, Grand Island, NY, USA) supplemented with 10% fetal bovine serum (FBS, Gibco, Grand Island, NY, USA) in a humidified incubator of 5% CO_2_ and 95% air at 37 °C. SH-SY5Y cells were obtained from the Institute of America’s type culture collection (ATCC, Manacas, VA, USA). Gas-miR36-5p mimics (5′-UGCAGAUGACUUGAUUUUGUUC-3′) and negative control (NC) mimics (5′-UUCUCCGAACGUGUCACGUTT-3′) were synthesized by Gene Pharma (Shanghai, China) and dissolved in RNA-free H_2_O with a final concentration of 20 μM. According to the instructions of the X-TremeGene siRNA Transfection Reagent (Roche, Basel, Switzerland), cells were seeded onto various cell culture plates, and the transfection reagent was mixed with serum-free DMEM in different proportions. After incubating at room temperature for 15 min, the mixture was evenly added to the cells for continuous culture for 24–48 h.

### 4.2. RNA Extraction and Real-Time Quantitative PCR

After the treatment, cell samples and mouse brain tissues were collected for total RNA extraction using Trizol (Takara, Kyoto, Japan). The primers used in this study were synthesized by Sangon (Shanghai, China) and are listed in Table 1. Subsequently, a cDNA reverse transcription kit (Toyobo, Osaka, Japan) was utilized for the reverse transcription process. General primers were employed for the reverse transcription of commonly expressed genes, while specifically designed stem-loop primers were used for the reverse transcription of *G. elata*-specific miRNAs. The conditions used during the reverse transcription are as follows: the samples were incubated at 42 °C for 15 min, followed by a heat shock at 85 °C for 5 s. Subsequently, they were maintained at 10 °C for long-term preservation. RT-qPCR was performed using the SYBR Green Master kit (Proteintech, Chicago, IL, USA) with an initial denaturation step at 95 °C for 10 min, followed by amplification cycles consisting of denaturation at 95 °C for 15 s and annealing/extension at 60 °C for 1 min. The final step included denaturation at 95 °C for 15 s.

The 5.8 s rRNA was used as the internal control for detecting the cellular content of *G. elata*-specific miRNA (Gas-miR36-5p). The expression level of the GSK-3β gene was normalized to GAPDH as a reference control. The relative gene expression level was determined using the ABI 7500 instrument (Applied Biosystems, Waltham, MA, USA) and analyzed with the 2^−ΔΔCt^ method.

### 4.3. Target Prediction and Dual-Luciferase Reporter Assay

To identify the specific *G. elata* miRNAs capable of interacting with GSK-3β mRNA (NM_001146156.2), we used three prediction software tools (miRmap, https://mirmap.ezlab.org/ (accessed on 10 April 2021); RNA22v2, https://cm.jefferson.edu/rna22/Interactive/ (accessed on 10 April 2021.); and RNAhybrid, https://bibiserv.cebitec.uni-bielefeld.de/rnahybrid/ (accessed on 10 April 2021). The candidate *G. elata* miRNA targeting GSK-3β was determined based on the matching method with the 3′-UTR and the binding energy. Subsequently, we designed site fragments corresponding to different binding sites of the candidate *G. elata*-specific miRNA and ligated them to the p-miRGLO double luciferase vector using the double luciferase kit (Gene Pharma, Shanghai, China) to generate recombinant plasmids, designated as GSK-3β-WT-pmiRGLO and GSK-3β-MUT-pmiRGLO (Figure 2A–C).

The SH-SY5Y cells were seeded in 6-well plates, and after 24 h, the two recombinant plasmids were co-transfected with NC mimics and Gas-miR36-5p into SH-SY5Y cells for 48–72 h, following the recommended ratio of X-tremeGene SiRNA Transfection Reagent. The follow-up process was conducted according to the double luciferase kit (Gene Pharma, Shanghai, China), and ultimately, the results were measured at wavelengths A560 and A465, respectively.

### 4.4. Protein Extraction and Western Blot

The AD cell model of SH-SY5Y cells transfected with Gas-miR36-5p mimics was maintained for 24 h, followed by induction with β-amyloid_25–35_ (Aβ_25–35_, Promega, Madison, WI, USA) at a concentration of 20 μM for an additional 24 h. Mouse brain tissues were collected, and total protein was extracted using an appropriate amount of radioimmunoprecipitation assay lysis buffer (RIPA, Beyotime, Nantong, China), supplemented with Phenylmethanesulfonyl fluoride (PMSF, Beyotime, Nantong, China) and a phosphatase inhibitor (Beyotime, Nantong, China). Protein quantification was performed prior to separation on a 12.5% SDS-PAGE protein gel. The isolated proteins were transferred to polyvinylidene fluoride membranes (PVDF, Merck, Darmstadt, Germany) by western blot. Subsequently, the membranes were blocked with 5% skimmed milk powder (prepared with TBST (pH 7.4)) for a duration of 2 h at room temperature. The membrane sealed with 5% skimmed milk was incubated overnight at 4 °C along with the following primary antibodies: GSK-3β (Proteintech, Chicago, IL, USA; cat. No. 67558-1-Ig, 1:5000), Total Tau (Proteintech, Chicago, IL, USA; cat. No. 66499-1-Ig, 1:1000), p-Tau Ser396 (Abcam, Cambridge, UK; cat. No. ab32057, 1:1000), p-Tau Thr181 (Abcam, Cambridge, UK; cat. No. ab254409, 1:1000), and GAPDH (Abcam, Cambridge, UK; cat. No. 60004-1-Ig, 1:4000). The subsequent PVDF membranes were washed with TBST. The corresponding secondary antibodies, HRP-labeled (horseradish peroxidase-labeled) goat anti-rabbit antibody (Proteintech, Chicago, IL, USA; cat. No. SA00001-2, 1:4000) and goat anti-mouse antibody (Proteintech, Chicago, IL, USA; cat. No. SA00001-1, 1:4000), were used as the secondary antibodies based on the species of different primary antibodies. Incubation with the secondary antibody was carried out for 2 h, followed by washing of the membrane with TBST for detection purposes. Protein bands were visualized using enhanced chemiluminescence (ECL, Beyotime, Nantong, China) performed with Tanon 5500 (Tanon 5500 Multi, Shanghai, China) to verify the results. Similarly, tissue samples from animals underwent identical treatment with an adjustment of RIPA lysate volume in proportion to tissue mass.

### 4.5. Animals and Treatments

Thirty male C57BL/6 mice weighing between 22 and 25 g were procured from Slacom (Shanghai, China). The fresh *G. elata* sample (from Shanxi, China) was authenticated by Professor Zongsuo Liang at the Zhejiang Provincial Key Laboratory of Plant Secondary Metabolism and Regulation. All animal handling and surgical procedures were approved by the Animal Ethics Review Committee of Zhejiang Sci-Tech University (Approval No. 202112016). The animals were housed in cages, containing five mice per cage, maintained under a 12-h light–dark cycle and at a room temperature of 24 °C. They had free access to adequate water and feed. The experimental mice were divided into a blank control group (NC), an Alzheimer’s disease model group (AD), a donepezil hydrochloride group (positive control, PC), and *G. elata* total RNA gavage groups with different doses (low, medium, and high doses). To induce the AD mouse model, we administered intraperitoneal injections of D-galactose (Solarbio, Beijing, China) at a dose of 150 mg/kg combined with gavage of aluminum trichloride (Macklin, Shanghai, China) (AlCl_3_) at a dose of 15 mg/kg for a duration of 40 days. In contrast, the blank control group received normal saline. Approximately 1 h after the modeling treatment, *G. elata* RNA groups (low, medium, and high dose groups) were intragastrically administered at different doses, maintaining the administration for 40 days. *G. elata* total RNA was administered intragastrically to mice at low (3 mg/kg), medium (6 mg/kg), and high (9 mg/kg) doses based on their body weight. Additionally, for the PC group, donepezil hydrochloride (Solarbio, Beijing, China) was administered intragastrically at a dosage of 3 mg/kg. The total RNA of *G. elata* was extracted using the Trizol method and subsequently dissolved in normal saline (Solarbio, Beijing, China). The weight fluctuations of the mice were monitored throughout the 40-day duration of the experiment.

### 4.6. Morris Water Maze Experiment

The platform (Sansbio, Nanjing, China) was positioned in a pool with a diameter of 1.2 m, elevated 0.5–1.5 cm above the water surface. A specific point at the pool’s edge was carefully chosen to release the mice into the water. Following release, only mice capable of swimming directly towards and onto the platform were selected for subsequent experiments. The mice were subjected to four daily training sessions, each of which had a fixed duration. The swimming pool was partitioned into four quadrants aligned with the cardinal directions (east, west, south, and north). At the midpoint of these quadrants, the mice were released into the water. Upon release, their heads faced towards the wall of the swimming pool. The swimming time and trajectory of the mice in locating the platform were recorded through an imaging system. In cases where the mice failed to locate the platform within 60 s, a score of 60 s was assigned, and they were guided to the platform for a subsequent resting period of 10 s during training. The results of each day were recorded as a mean value of four repetitions. In case the mice jumped off the platform during rest, the guidance was repeated and the timer was reset until a duration of 10 s was achieved. The final score represented the average performance throughout the entire incubation period. The platform was removed from the pool 24 h after the positioning navigation test. Any water entry point was selected, and the exploration path of mice in the swimming pool within a duration of 120 s was recorded by the imaging system. The video data obtained from both the mouse positioning navigation experiment and the space exploration experiment were processed and analyzed by the software Image J Animal Tracker (V 1.53J).

### 4.7. Statistical Analysis

The data presented in this study are the average of three separate experiments, expressed as the mean ± SEM. Data analysis was performed using GraphPad Prism 8.0 software, and results were reported as mean ± standard deviation (SD). Statistical analyses included a two-tailed Student’s *t*-test, a one-way ANOVA, and Pearson’s correlation coefficient. A statistically significant difference was considered at *p* < 0.05.

## 5. Conclusions

In this study, we initially confirmed through bioinformatics analysis that GSK-3β is a target of Gas-miR36-5p, a unique component derived from *G. elata*. Subsequently, we assessed the expression of GSK-3β at both the transcriptional and post-translational levels, revealing that Gas-miR36-5p can downregulate GSK-3β expression at the post-translational level. We then investigated the regulatory effects of the *G. elata*-specific miRNA, Gas-miR36-5p, on the AD-associated GSK-3β signaling pathway at both in vitro and in vivo levels, respectively. We confirmed the possible transfection of Gas-miR36-5p into SH-SY5Y cells and detected its presence in mouse brain tissue through gavage administration.

Our in vitro experiments demonstrated that Gas-miR36-5p effectively suppressed the expression of GSK-3β in the AD cell model and inhibited hyperphosphorylation of Tau. Simultaneously, it ameliorated the related oxidative stress indicators associated with the AD cell model. In vivo experiments, including the Morris water maze test and behavioral assessments of mice after various treatments, revealed a gradual decrease in escape latency during the place navigation test in AD mouse models with increasing intragastric concentrations of *G. elata* total RNA. Additionally, there was an observed upward trend in effective exploration time and the number of target-platform crossings during the spatial exploration test. As the intragastric concentration of *G. elata* total RNA increased in the low/medium/high-dose groups, a corresponding decrease was noted in intracerebral GSK-3β levels among AD mice, accompanied by significant reductions in Tau protein phosphorylation levels at the Thr181 and Ser396 sites. Moreover, the oxidative stress indicators in the brain tissue of AD mice exhibited varying degrees of improvement. These findings suggest that Gas-miR36-5p or the regulation of GSK-3β in the brains of AD mice can effectively inhibit Tau hyperphosphorylation, providing preliminary evidence for the neuroprotective effects of *G. elata* RNA and its ability to enhance learning and memory abilities in AD mice.

In conclusion, our experiments have yielded preliminary insights into the molecular mechanism underlying Gas-miR36-5p’s ability to mitigate Tau hyperphosphorylation through targeted inhibition of GSK-3β activity. Additionally, we have substantiated the prophylactic and therapeutic potential of *G. elata* RNA in Alzheimer’s disease (AD). These findings establish a foundation for comprehending the regulatory effects exerted by *G. elata*-specific miRNA as an innovative active component in AD therapy, thereby providing novel perspectives for AD prevention and management utilizing *G. elata*.

## Figures and Tables

**Table 1:** Primer sequences used for reverse transcription and RT-qPCR.

Gene		Primer Sequence (5′-3′)	Application
Gas-miR36-5p	Loop-stem primer	CTCAACTGGTGTCGTGGAGTCGGCAATTCAGTTGAGCGAACAAAAT	Reverse transcription
	Forward	GCCGAGTGCAGATGACTTG	qRT-PCR
	Reverse	CTCAACTGGTGTCGTGGA	
5.8 s rRNA	Forward	ATCACTCGGCTCGTGCGTC	qRT-PCR
	Reverse	CAAGTGCGTTCGAAGTGTCG	
GSK-3β	Forward	AGGAGAACCCAATGTTTCGTAT	qRT-PCR
	Reverse	ATCCCCTGGAAATATTGGTTGT	
GAPDH	Forward	AATGGATTTGGACGCATTGGT	qRT-PCR
	Reverse	TTTGCACTGGTACGTGTTGAT	

## Data Availability

The data that support the results of this study are available within the article and the Supporting Information. Further data are available from the corresponding author upon reasonable request.

## Supplementary Materials

The supporting information can be downloaded at: https://www.mdpi.com/article/10.3390/ijms242417295/s1.

## References

[1] Qiu C., Kivipelto M., Von Strauss E. (2009). Epidemiology of alzheimer’s disease: Occurrence, determinants, and strategies toward intervention. Dialogues Clin. Neurosci..

[2] Richard H. (2018). Alzheimer’s disease. Nature.

[3] Guzman-Martinez L., Calfío C., Farias G.A., Vilches C., Prieto R., Maccioni R.B. (2021). New frontiers in the prevention, diagnosis, and treatment of alzheimer’s disease. J. Alzheimer’s Dis..

[4] Carter M.D., Simms G.A., Weaver D.F. (2010). The development of new therapeutics for alzheimer’s disease. Clin. Pharmacol. Ther..

[5] Thal D.R., Walter J., Saido T.C., Fändrich M. (2015). Neuropathology and biochemistry of aβ and its aggregates in alzheimer’s disease. Acta Neuropathol..

[6] Alonso A.D.C., Grundke-Iqbal I., Iqbal K. (1996). Alzheimer’s disease hyperphosphorylated tau sequesters normal tau into tangles of filaments and disassembles microtubules. Nat. Med..

[7] Wen Y., Planel E., Herman M., Figueroa H.Y., Wang L., Liu L., Lau L.F., Yu W.H., Duff K.E. (2008). Duff. Interplay between cyclin-dependent kinase 5 and glycogen synthase kinase 3 beta mediated by neuregulin signaling leads to differential effects on tau phosphorylation and amyloid precursor protein processing. J. Neurosci..

[8] Tanaka M., Szabó Á., Vécsei L., Giménez-Llort L. (2023). Emerging Translational Research in Neurological and Psychiatric Diseases: From In Vitro to In Vivo Models. Int. J. Mol. Sci..

[9] Busche M.A., Konnerth A. (2016). Impairments of neural circuit function in Alzheimer’s disease. Philos. Trans. R. Soc. B Biol. Sci..

[10] Harris S.S., Wolf F., De Strooper B., Busche M.A. (2020). Tipping the Scales: Peptide-Dependent Dysregulation of Neural Circuit Dynamics in Alzheimer’s Disease. Neuron.

[11] Ranasinghe K.G., Verma P., Cai C., Xie X., Kudo K., Gao X., Lerner H., Mizuiri D., Strom A., Iaccarino L. (2022). Altered excitatory and inhibitory neuronal subpopulation parameters are distinctly associated with tau and amyloid in Alzheimer’s disease. eLife.

[12] Martin L., Latypova X., Wilson C.M., Magnaudeix A., Terro F. (2012). Tau protein kinases: Involvement in alzheimer’s disease. Ageing Res. Rev..

[13] Laurent C., Buée L., Blum D. (2018). Tau and neuroinflammation: What impact for alzheimer’s disease and tauopathies?. Biomed. J..

[14] Liu S.J., Zhang J.Y., Li H.L., Fang Z.Y., Wang Q., Deng H.M., Gong C.X., Grundke-Iqbal I., Iqbal K., Wang J.Z. (2004). Tau becomes a more favorable substrate for GSK-3 when it is prephosphorylated by PKA in rat brain. J. Biol. Chem..

[15] Zhou Q., Wang M., Du Y., Zhang W., Bai M., Zhang Z., Li Z., Miao J. (2015). Inhibition of c-Jun N-terminal kinase activation reverses Alzheimer disease phenotypes in APPswe/PS1dE9 mice. Ann. Neurol..

[16] Munoz L., Ammit A.J. (2010). Targeting p38 MAPK pathway for the treatment of Alzheimer’s disease. Neuropharmacology.

[17] Chen D., Lan G., Li R., Mei Y., Shui X., Gu X., Wang L., Zhang T., Gan C.L., Xia Y. (2022). Melatonin ameliorates tau-related pathology via the miR-504-3p and CDK5 axis in Alzheimer’s disease. Transl. Neurodegener..

[18] Martinez A., Perez D.I. (2008). GSK-3 inhibitors: A ray of hope for the treatment of Alzheimer’s disease?. J. Alzheimer’s Dis..

[19] Duda P., Akula S.M., Abrams S.L., Steelman L.S., Mccubrey J.A. (2020). Targeting gsk3 and associated signaling pathways involved in cancer. Cells.

[20] Bai F., Shi Y., Yuan Y., Yue C., Zhuang L., Xu X., Liu X., Zhang Z. (2012). Association of a GSK-3β polymorphism with brain resting-state function in amnestic-type mild cognitive impairment. J. Alzheimer’s Dis..

[21] Ishiguro K., Shiratsuchi A., Sato S., Omori A., Imahori K. (1993). Glycogen synthase kinase 3 beta is identical to tau protein kinase i generating several epitopes of paired helical filaments. FEBS Lett..

[22] Chen C.H., Zhou W., Liu S., Deng Y., Cai F., Tone M., Tone Y., Tong Y., Song W. (2012). Increased nf-kb signalling up-regulates bace1 expression and its therapeutic potential in alzheimer’s disease. Int. J. Neuropsychopharmacol..

[23] Liu F., Liang Z., Shi J., Yin D., El-Akkad E., Grundke-Iqbal I., Iqbal K., Gong C.X. (2006). Pka modulates gsk-3beta- and cdk5-catalyzed phosphorylation of tau in site- and kinase-specific manners. FEBS. Lett..

[24] Qi Z., Zhang Y., Yao K., Zhang M., Xu Y., Zhang J., Bai X., Zu H. (2021). DHCR24 Knockdown Lead to Hyperphosphorylation of Tau at Thr181, Thr231, Ser262, Ser396, and Ser422 Sites by Membrane Lipid-Raft Dependent PP2A Signaling in SH-SY5Y Cells. Neurochem. Res..

[25] Sayas C.L., Ávila J. (2021). GSK-3 and Tau: A Key Duet in Alzheimer’s Disease. Cells.

[26] Lucas J.J., Hernández F., Gómez-Ramos P., Morán M.A., Hen R., Avila J. (2001). Decreased nuclear beta-catenin, tau hyperphosphorylation and neurodegeneration in gsk-3beta conditional transgenic mice. EMBO J..

[27] Qu C., Li Q.P., Su Z.R., Ip S.P., Yuan Q.J., Xie Y.L., Xu Q.Q., Yang W., Huang Y.F., Xian Y.F. (2022). Nano-honokiol ameliorates the cognitive deficits in tgcrnd8 mice of alzheimer’s disease via inhibiting neuropathology and modulating gut microbiota. J. Adv. Res..

[28] Kaidanovich Beilin O., Lipina T.V., Takao K., van Eede M., Hattori S., Laliberté C., Khan M., Okamoto K., Chambers J.W., Fletcher P.J. (2009). Abnormalities in brain structure and behavior in gsk-3alpha mutant mice. Mol. Brain.

[29] Liu J., Zuo X., Han J., Dai Q., Xu H., Liu Y., Cui S. (2020). Mir-9-5p inhibits mitochondrial damage and oxidative stress in ad cell models by targeting gsk-3β. Biosci. Biotechnol. Biochem..

[30] Jiang H., Liu J., Guo S., Zeng L., Cai Z., Zhang J., Wang L., Li Z., Liu R. (2022). MiR-23b-3p rescues cognition in Alzheimer’s disease by reducing tau phosphorylation and apoptosis via GSK-3β signaling pathways. Mol. Ther.-Nucleic Acids.

[31] Kou X., Chen D., Chen N. (2020). The Regulation of microRNAs in Alzheimer’s Disease. Front. Neurol..

[32] Avila J., Hernández F. (2007). Gsk-3 inhibitors for alzheimer’s disease. Expert Rev. Neurother..

[33] Xu Y., Wang H., Zhang J.G. (2020). Role and Biological Significance of GSK-3β Signal Pathway in Alzheimer’s Disease. Her. Med..

[34] Rippin I. (2021). Mechanisms and therapeutic implications of gsk-3 in treating neurodegeneration. Cells.

[35] Zhang B., Wang Q., Pan X., Zhang B., Wang Q., Pan X. (2007). Micrornas and their regulatory roles in animals and plants. J. Cell. Physiol..

[36] Ambros V. (2004). The functions of animal microRNAs. Nature.

[37] Filipowicz W., Jaskiewicz L., Kolb F.A., Pillai R.S. (2005). Post-transcriptional gene silencing by siRNAs and miRNAs. Curr. Opin. Struct. Biol..

[38] Sun M., Xu S., Mei Y., Li J., Gu Y., Zhang W., Wang J. (2022). MicroRNAs in medicinal plants. Int. J. Mol. Sci..

[39] Wang W., Liu D., Zhang X., Chen D., Cheng Y., Shen F. (2018). Plant microRNAs in cross-kingdom regulation of gene expression. Int. J. Mol. Sci..

[40] Zhang L., Hou D., Chen X., Li D., Zhu L., Zhang Y., Li J., Bian Z., Liang X., Cai X. (2012). Exogenous plant MIR168a specifically targets mammalian LDLRAP1: Evidence of cross-kingdom regulation by microRNA. Cell Res..

[41] Zhou Z., Li X., Liu J., Dong L., Chen Q., Liu J., Kong H., Zhang Q., Qi X., Hou D. (2015). Honeysuckle-encoded atypical microrna2911 directly targets influenza a virus. Cell Res..

[42] Chin A.R., Fong M.Y., Somlo G., Wu J., Swiderski P., Wu X., Wang S.E. (2016). Cross-kingdom inhibition of breast cancer growth by plant miR159. Cell Res..

[43] Yuan L., Jialiang G., Min P., Hongyan M., Hongbo M., Pingping C., Yuan X., Qiong Z., Guomin S. (2018). A review on central nervous system effects of gastrodin. Front. Pharmacol..

[44] Ojemann L.M., Nelson W.L., Shin D.S., Rowe A.O., Buchanan R.A. (2006). Tian ma, an ancient chinese herb, offers new options for the treatment of epilepsy and other conditions. Epilepsy Behav..

[45] Xia C., Zhou H., Xu X., Jiang T., Li S., Wang D., Nie Z., Sheng Q. (2020). Identification and investigation of miRNAs from *Gastrodia elata* blume and their potential function. Front. Pharmacol..

[46] Wang M., Qin L., Tang B. (2019). Micrornas in alzheimer’s disease. Front. Genet..

[47] Sun X., Deng Y., Ge P., Peng Q., Soufiany I., Zhu L., Duan R. (2023). Diminazene ameliorates neuroinflammation by suppression of astrocytic mirna-224-5p/nlrp3 axis in alzheimer’s disease model. J. Inflamm. Res..

[48] Xu J., Wu K.J., Jia Q.J., Ding X.F. (2020). Roles of miRNA and lncRNA in triple-negative breast cancer. J. Zhejiang Univ. Sci. B.

[49] Xuan C., Yang E., Zhao S., Xu J., Li P., Zhang Y., Jiang Z., Ding X. (2023). Regulation of LncRNAs and microRNAs in neuronal development and disease. PeerJ..

[50] Wei B., Huang B., Zhao X. (2023). An overview of biochemical technologies for the cancer biomarker miR-21 detection. Anal. Sci..

[51] Gao Y., Feng B., Gao C., Zhang H., Wen F., Tao L., Fu G., Xiong J. (2022). The Evolution and Functional Roles of miR408 and Its Targets in Plants. Int. J. Mol. Sci..

[52] Mei J., Wu Y., Niu Q., Miao M., Zhang D., Zhao Y., Cai F., Yu D., Ke L., Feng H. (2022). Integrative Analysis of Expression Profiles of mRNA and MicroRNA Provides Insights of Cotton Response to *Verticillium dahliae*. Int. J. Mol. Sci..

[53] Xu W., Fan H., Pei X., Hua X., Xu T., He Q. (2023). mRNA-Seq and miRNA-Seq Analyses Provide Insights into the Mechanism of *Pinellia ternata* Bulbil Initiation Induced by Phytohormones. Genes.

[54] Chi X., Wang Z., Wang Y., Liu Z., Wang H., Xu B. (2023). Cross-kingdom regulation of plant-derived mirnas in modulating insect development. Int. J. Mol. Sci..

[55] Rabuma T., Gupta O.P., Chhokar V. (2022). Recent advances and potential applications of cross-kingdom movement of mirnas in modulating plant’s disease response. RNA Biol..

[56] Kumar D., Kumar S., Ayachit G., Bhairappanavar S.B., Ansari A., Sharma P., Soni S., Das J. (2017). Cross-kingdom regulation of putative mirnas derived from happy tree in cancer pathway: A systems biology approach. Int. J. Mol. Sci..

[57] Yang J., Hotz T., Broadnax L., Yarmarkovich M., Elbaz-Younes I., Hirschi K.D. (2016). Anomalous uptake and circulatory characteristics of the plant-based small RNA MIR2911. Sci. Rep..

[58] Hardy J., Selkoe D.J. (2002). The amyloid hypothesis of Alzheimer’s disease: Progress and problems on the road to therapeutics. Science.

[59] Bloom G.S. (2014). Amyloid-β and tau: The trigger and bullet in Alzheimer disease pathogenesis. JAMA Neurol..

[60] Li S., Zhang B. (2013). Traditional Chinese medicine network pharmacology: Theory, methodology and application. Chin. J. Nat. Med..

[61] Zhan H.D., Zhou H.Y., Sui Y.P., Du X.L., Wang W.H., Dai L., Sui F., Huo H.R., Jiang T.L. (2016). The rhizome of *Gastrodia elata* Blume—An ethnopharmacological review. J. Ethnopharmacol..

