# Supplementary material for: Neuroprotection and Mechanism of Gas-miR36-5p from Gastrodia elata in an Alzheimer’s Disease Model by Regulating Glycogen Synthase Kinase-3β
Source: Int J Mol Sci. 2023 Dec 9;24(24):17295. doi: 10.3390/ijms242417295 (PMC10744203; doi:10.3390/ijms242417295)
Supplement: Supplementary file 1 [file ijms-24-17295-s001.zip › Datasheets of Tau related primary antibody/Abcam p-Tau Ser396 32057 Datasheet.pdf]

abcam

Product datasheet

Anti-Tau (phospho S396) antibody [E178] ab32057

Recombinant RabMAb

★★★★★ 5 Abreviews 60 References 15 Images

Overview

**Product name**	Anti-Tau (phospho S396) antibody [E178]
**Description**	Rabbit monoclonal [E178] to Tau (phospho S396)
**Host species**	Rabbit
**Specificity**	The specificity of this antibody refers to P10636-8.
**Tested applications**	**Suitable for:** IHC-P, Dot blot, ELISA, IHC-Fr, WB, IP<br>**Unsuitable for:** Flow Cyt
**Species reactivity**	**Reacts with:** Mouse, Rat, Human<br>**Predicted to work with:** Cow
**Immunogen**	Synthetic peptide. This information is proprietary to Abcam and/or its suppliers.
**Positive control**	WB: Human and mouse brain tissue lysates; IP: Human fetal brain lysates; IHC-P: Human cerebrum and salivary gland; Mouse colon and rat colon and tongue tissue; IHC-Fr: Mouse and Rat cerebrum tissue, Human Alzheimer brain tissue
**General notes**	This product is a recombinant monoclonal antibody, which offers several advantages including:<br>- High batch-to-batch consistency and reproducibility<br>- Improved sensitivity and specificity<br>- Long-term security of supply<br>- Animal-free production<br>For more information **see here**.<br>Our RabMAb® technology is a patented hybridoma-based technology for making rabbit monoclonal antibodies. For details on our patents, please refer to **RabMAb® patents**.<br>**We are constantly working hard to ensure we provide our customers with best in class antibodies. As a result of this work we are pleased to now offer this antibody in purified format. We are in the process of updating our datasheets. The purified format is designated 'PUR' on our product labels. If you have any questions regarding this update, please contact our Scientific Support team.**

Properties

**Form**	Liquid
**Storage instructions**	Shipped at 4°C. Upon delivery aliquot and store at -20°C. Avoid freeze / thaw cycles.

Dissociation constant (K_D) $K_D = 2.08 \times 10^{-11}$ M

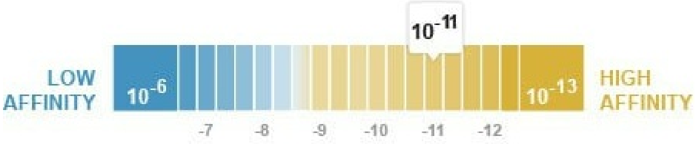

Learn more about K_D

Storage buffer pH: 7.20
Preservative: 0.01% Sodium azide
Constituents: 59% PBS, 40% Glycerol, 0.05% BSA

Purity Protein A purified

Clonality Monoclonal

Clone number E178

Isotype IgG

Applications

The Abpromise guarantee Our **Abpromise guarantee** covers the use of ab32057 in the following tested applications.

The application notes include recommended starting dilutions; optimal dilutions/concentrations should be determined by the end user.

Application	Abreviews	Notes
**IHC-P**	★★★★★ (1)	1/1000 - 1/4000. Perform heat mediated antigen retrieval with Tris/EDTA buffer pH 9.0 before commencing with IHC staining protocol.<br>We do not guarantee IHC-P for mouse and rat. The mouse and rat recommendation is based on other applications.
**AP**		Use at an assay dependent concentration.<br>**Antibody concentration range -** 3.33, 1.67, 0.83, 0.42, 0.21, 0 nM/mL
**Dot blot**		1/1000.
**ELISA**		Use at an assay dependent concentration.
**IHC-Fr**	★★★★★ (1)	1/500.<br>Heat mediated antigen retrieval using sodium citrate buffer (10mM citrate pH 6.0 + 0.05% Tween-20)
**WB**	★★★★★ (2)	1/1000. Predicted molecular weight: 79 kDa.<br>**For unpurified use at 1/5000**
**IP**		1/20. **For unpurified use at 1/100**

Application notes Is unsuitable for Flow Cyt.

Target

**Function**	Promotes microtubule assembly and stability, and might be involved in the establishment and maintenance of neuronal polarity. The C-terminus binds axonal microtubules while the N-terminus binds neural plasma membrane components, suggesting that tau functions as a linker protein between both. Axonal polarity is predetermined by tau localization (in the neuronal cell) in the domain of the cell body defined by the centrosome. The short isoforms allow plasticity of the cytoskeleton whereas the longer isoforms may preferentially play a role in its stabilization.
**Tissue specificity**	Expressed in neurons. Isoform PNS-tau is expressed in the peripheral nervous system while the others are expressed in the central nervous system.
**Involvement in disease**	Note=In Alzheimer disease, the neuronal cytoskeleton in the brain is progressively disrupted and replaced by tangles of paired helical filaments (PHF) and straight filaments, mainly composed of hyperphosphorylated forms of TAU (PHF-TAU or AD P-TAU).<br>Defects in MAPT are a cause of frontotemporal dementia (FTD) [MIM:600274]; also called frontotemporal dementia (FTD), pallido-ponto-nigral degeneration (PPND) or historically termed Pick complex. This form of frontotemporal dementia is characterized by presenile dementia with behavioral changes, deterioration of cognitive capacities and loss of memory. In some cases, parkinsonian symptoms are prominent. Neuropathological changes include frontotemporal atrophy often associated with atrophy of the basal ganglia, substantia nigra, amygdala. In most cases, protein tau deposits are found in glial cells and/or neurons.<br>Defects in MAPT are a cause of Pick disease of the brain (PIDB) [MIM:172700]. It is a rare form of dementia pathologically defined by severe atrophy, neuronal loss and gliosis. It is characterized by the occurrence of tau-positive inclusions, swollen neurons (Pick cells) and argentophilic neuronal inclusions known as Pick bodies that disproportionally affect the frontal and temporal cortical regions. Clinical features include aphasia, apraxia, confusion, anomia, memory loss and personality deterioration.<br>Note=Defects in MAPT are a cause of corticobasal degeneration (CBD). It is marked by extrapyramidal signs and apraxia and can be associated with memory loss. Neuropathologic features may overlap Alzheimer disease, progressive supranuclear palsy, and Parkinson disease.<br>Defects in MAPT are a cause of progressive supranuclear palsy type 1 (PSNP1) [MIM:601104, 260540]; also abbreviated as PSP and also known as Steele-Richardson-Olszewski syndrome. PSNP1 is characterized by akinetic-rigid syndrome, supranuclear gaze palsy, pyramidal tract dysfunction, pseudobulbar signs and cognitive capacities deterioration. Neurofibrillary tangles and gliosis but no amyloid plaques are found in diseased brains. Most cases appear to be sporadic, with a significant association with a common haplotype including the MAPT gene and the flanking regions. Familial cases show an autosomal dominant pattern of transmission with incomplete penetrance; genetic analysis of a few cases showed the occurrence of tau mutations, including a deletion of Asn-613.
**Sequence similarities**	Contains 4 Tau/MAP repeats.
**Developmental stage**	Four-repeat (type II) tau is expressed in an adult-specific manner and is not found in fetal brain, whereas three-repeat (type I) tau is found in both adult and fetal brain.
**Domain**	The tau/MAP repeat binds to tubulin. Type I isoforms contain 3 repeats while type II isoforms contain 4 repeats.
**Post-translational modifications**	Phosphorylation at serine and threonine residues in S-P or T-P motifs by proline-directed protein kinases (PDPK: CDK1, CDK5, GSK-3, MAPK) (only 2-3 sites per protein in interphase, seven-fold increase in mitosis, and in PHF-tau), and at serine residues in K-X-G-S motifs by MAP/microtubule affinity-regulating kinase (MARK) in Alzheimer diseased brains. Phosphorylation decreases with age. Phosphorylation within tau's repeat domain or in flanking regions seems to reduce tau's interaction with, respectively, microtubules or plasma membrane components. Phosphorylation on Ser-610, Ser-622, Ser-641 and Ser-673 in several isoforms during mitosis.

Polyubiquitinated. Requires functional TRAF6 and may provoke SQSTM1-dependent degradation by the proteasome (By similarity). PHF-tau can be modified by three different forms of polyubiquitination. 'Lys-48'-linked polyubiquitination is the major form, 'Lys-6'-linked and 'Lys-11'-linked polyubiquitination also occur.
Glycation of PHF-tau, but not normal brain tau. Glycation is a non-enzymatic post-translational modification that involves a covalent linkage between a sugar and an amino group of a protein molecule forming ketoamine. Subsequent oxidation, fragmentation and/or cross-linking of ketoamine leads to the production of advanced glycation endproducts (AGES). Glycation may play a role in stabilizing PHF aggregation leading to tangle formation in AD.

Cellular localization

Cytoplasm > cytosol. Cell membrane. Cytoplasm > cytoskeleton. Cell projection > axon. Mostly found in the axons of neurons, in the cytosol and in association with plasma membrane components.

Form

There are 9 isoforms produced by alternative splicing.

Images

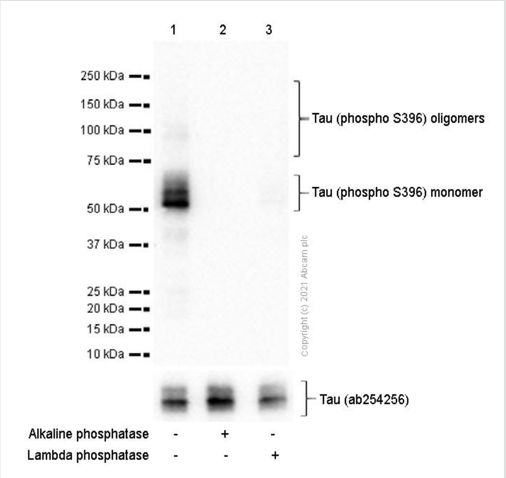

Western blot - Anti-Tau (phospho S396) antibody [E178] (ab32057)

All lanes : Anti-Tau (phospho S396) antibody [E178] (ab32057) at 1/1000 dilution

Lane 1 : Human brain lysates
Lane 2 : Human brain lysates and the membrane was incubated with alkaline phosphatase
Lane 3 : Human brain lysates and the membrane was incubated with lambda phosphatase

Lysates/proteins at 15 µg per lane.

Secondary
All lanes : Goat Anti-Rabbit IgG (HRP) with minimal cross-reactivity with human IgG at 1/2000 dilution

Predicted band size: 79 kDa
Observed band size: 50-79 kDa

Exposure time: 60 seconds

Blocking/Diluting buffer and concentration 5% NFDM/TBST

Tau assembles into oligomers as described in PMID: 28382304, 32692785 and 30120733.

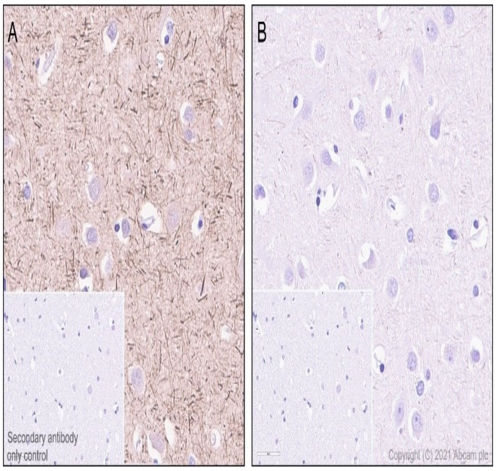

Immunohistochemistry (Formalin/PFA-fixed paraffin-embedded sections) - Anti-Tau (phospho S396) antibody [E178] (ab32057)

Immunohistochemistry analysis of paraffin-embedded human cerebrum tissue sections labeling Tau (phospho S396) with ab32057 at 1/4000 dilution (0.026 μg/mL). Goat Anti-Rabbit IgG H&L (HRP polymer) was used as the secondary antibody. Sections were counterstained with Hematoxylin. Antigen retrieval was heat mediated using **ab93684** (Tris/EDTA buffer, pH 9.0).

Positive staining on human cerebrum without alkaline phosphatase treatment (image A). No staining on human cerebrum with alkaline phosphatase treatment (image B).
The section was incubated with ab32057 overnight at +4°C.

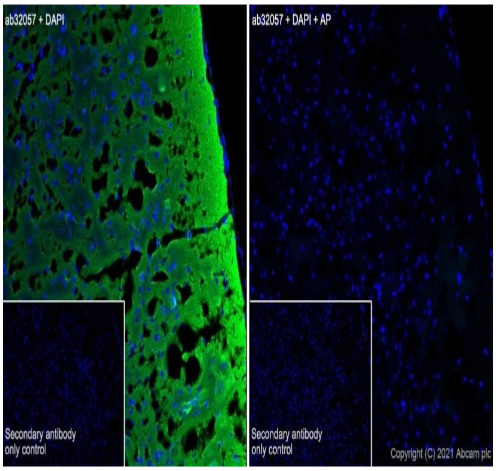

Immunohistochemistry (Frozen sections) - Anti-Tau (phospho S396) antibody [E178] (ab32057)

Immunohistochemistry analysis of frozen mouse cerebrum tissue sections labeling Tau (phospho S396) with ab32057 at 1/100 (1μg/mL). **ab150077** AlexaFluor® 488 Goat anti-Rabbit at 1/1000 (2 μg/mL) was used as the secondary antibody. Sections were fixed with 4% PFA and permeabilised with 0.2% Triton X-100. DAPI (blue) was used as nuclear counterstain. Antigen retrieval was heat mediated using sodium citrate buffer (10mM citrate pH 6.0 + 0.05% Tween-20).

Cytoplasmic staining on mouse cerebrum, the signal decreased after phosphatase treatment at 37l for 2h.

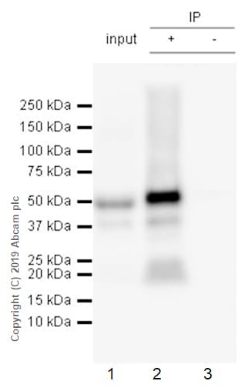

Immunoprecipitation - Anti-Tau (phospho S396) antibody [E178] (ab32057)

ab32057 (purified) at 1/20 dilution (0.5ug) immunoprecipitating Tau in Human fetal brain lysates.

Lane 1: Human fetal brain lysates 10ug

Lane 2 (+): ab32057 & Human fetal brain lysates

Lane 3 (-): Rabbit monoclonal IgG (**ab172730**) instead of ab32057 in Human fetal brain lysates

For western blotting, VeriBlot for IP Detection Reagent (HRP) (**ab131366**) was used at 1/1000 dilution.

Blocking and diluting buffer: 5% NFDM/TBST.

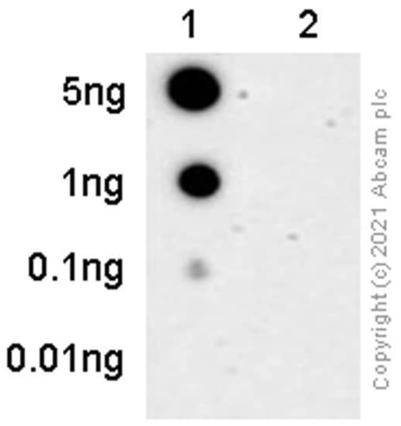

Dot Blot - Anti-Tau (phospho S396) antibody [E178] (ab32057)

Dot blot analysis of Tau (phospho S396) phospho peptide (Lane 1) and Tau non-phospho peptide (Lane 2) labelling Tau (phospho S396) with ab32057 at a dilution of 1/1000. **ab97051** (Peroxidase conjugated goat anti-rabbit IgG (H+L)) was used as the secondary antibody at a dilution of 1/100000.

Blocking and dilution buffer: 5% NFDM/TBST.

Exposure time: 3 minutes.

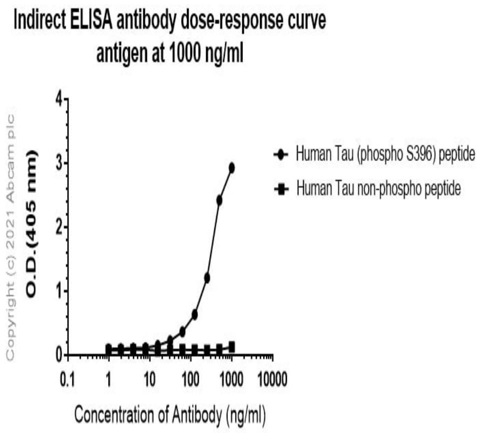

ELISA - Anti-Tau (phospho S396) antibody [E178] (ab32057)

Indirect ELISA antigen dose-response curve using ab32057 at 1000-0 ng/mL. Antigen Human Tau (phospho S396) peptide, Human Tau non-phospho peptide at concentration of 1000 ng/mL. Alkaline Phosphatase-conjugated AffiniPure Goat Anti-Rabbit IgG H+L at 1/2500 dilution was used as the secondary antibody.

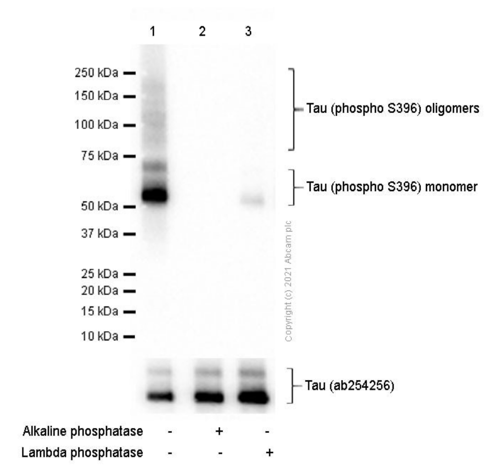

Western blot - Anti-Tau (phospho S396) antibody [E178] (ab32057)

All lanes : Anti-Tau (phospho S396) antibody [E178] (ab32057) at 1/1000 dilution

Lane 1 : Mouse brain lysates
Lane 2 : Mouse brain lysates and the membrane was incubated with alkaline phosphatase
Lane 3 : Mouse brain lysates and the membrane was incubated with lambda phosphatase

Lysates/proteins at 15 µg per lane.

Secondary
All lanes : Goat Anti-Rabbit IgG (HRP) with minimal cross-reactivity with human IgG at 1/2000 dilution

Predicted band size: 79 kDa
Observed band size: 50-79 kDa

Exposure time: 10 seconds

Blocking/Diluting buffer and concentration 5% NFDM/TBST

Tau assembles into oligomers as described in PMID: 28382304, 32692785 and 30120733.

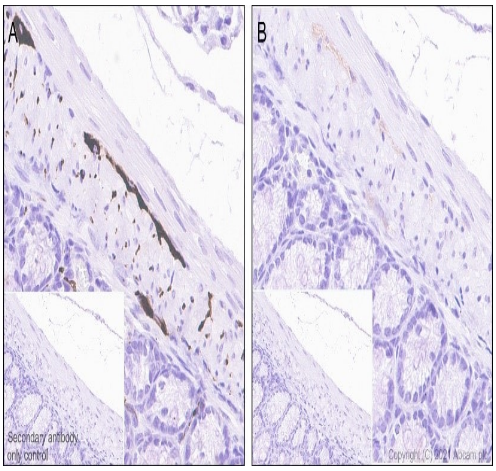

Immunohistochemistry (Formalin/PFA-fixed paraffin-embedded sections) - Anti-Tau (phospho S396) antibody [E178] (ab32057)

Immunohistochemistry analysis of paraffin-embedded mouse colon tissue sections labeling Tau (phospho S396) with ab32057 at 1/4000 dilution (0.026 µg/mL). Goat Anti-Rabbit IgG H&L (HRP polymer) was used as the secondary antibody. Sections were counterstained with Hematoxylin. Antigen retrieval was heat mediated using **ab93684** (Tris/EDTA buffer, pH 9.0).

Positive staining on ganglions of mouse colon without alkaline phosphatase treatment (image A). No staining on ganglions of mouse colon with alkaline phosphatase treatment (image B). The section was incubated with ab32057 overnight at +4°C.

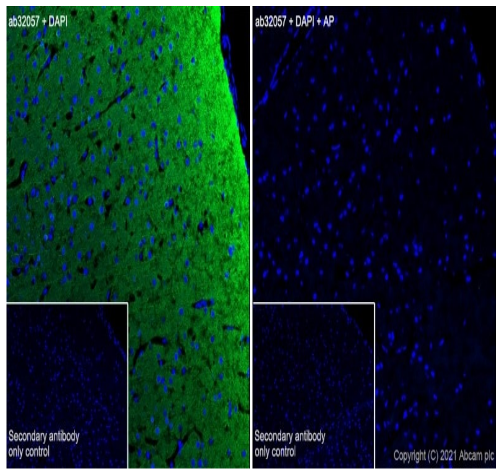

Immunohistochemistry (Frozen sections) - Anti-Tau (phospho S396) antibody [E178] (ab32057)

Immunohistochemistry analysis of frozen rat cerebrum tissue sections labeling Tau (phospho S396) with ab32057 at 1/100 (1 μg/mL). **ab150077** AlexaFluor®488 Goat anti-Rabbit at 1/1000 (2 μg/mL) was used as the secondary antibody. Sections were fixed with 4% PFA and permeabilised with 0.2% Triton X-100. DAPI (blue) was used as nuclear counterstain. Antigen retrieval was heat mediated using sodium citrate buffer (10mM citrate pH 6.0 + 0.05% Tween-20).

Cytoplasmic staining on rat cerebrum, the signal decreased after phosphatase treatment at 37I for 2h.

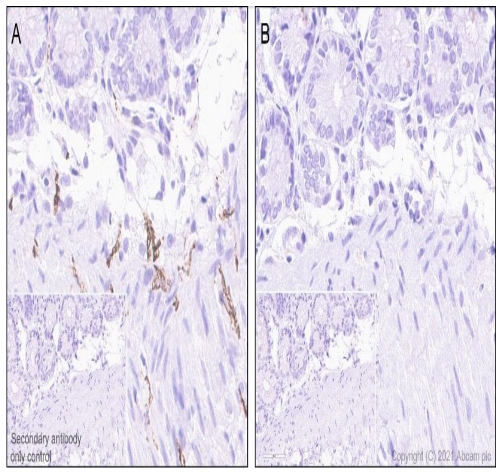

Immunohistochemistry (Formalin/PFA-fixed paraffin-embedded sections) - Anti-Tau (phospho S396) antibody [E178] (ab32057)

Immunohistochemistry analysis of paraffin-embedded rat colon tissue sections labeling Tau (phospho S396) with ab32057 at 1/4000 dilution (0.026 μg/mL). Goat Anti-Rabbit IgG H&L (HRP polymer) was used as the secondary antibody. Sections were counterstained with Hematoxylin. Antigen retrieval was heat mediated using **ab93684** (Tris/EDTA buffer, pH 9.0).

Positive staining on ganglions of rat colon without alkaline phosphatase treatment (image A). No staining on ganglions of rat colon with alkaline phosphatase treatment (image B).
The section was incubated with ab32057 overnight at +4°C.

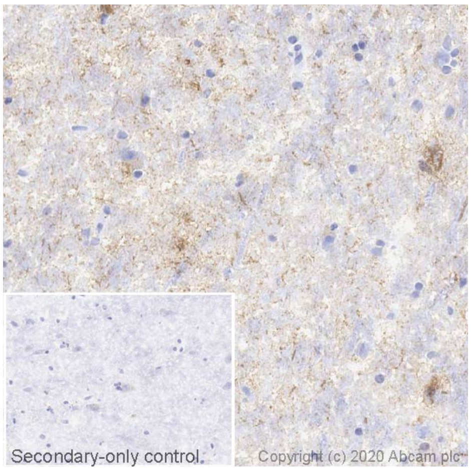

Immunohistochemistry (Frozen sections) - Anti-Tau (phospho S396) antibody [E178] (ab32057)

IHC image of Tau staining in a section of frozen normal human Alzheimer brain performed on a Leica BOND^{TM} system using the standard protocol. The section was fixed in 10% paraformaldehyde (10 min) prior to staining. The section was incubated with ab32057, 1/1000 dilution, for 15 mins at room temperature and detected using an HRP conjugated compact polymer system. DAB was used as the chromogen. The section was then counterstained with haematoxylin and mounted with DPX. The inset secondary-only control image is taken from an identical assay without primary antibody.

For other IHC staining systems (automated and non-automated) customers should optimize variable parameters such as antigen retrieval conditions, primary antibody concentration and antibody incubation times.

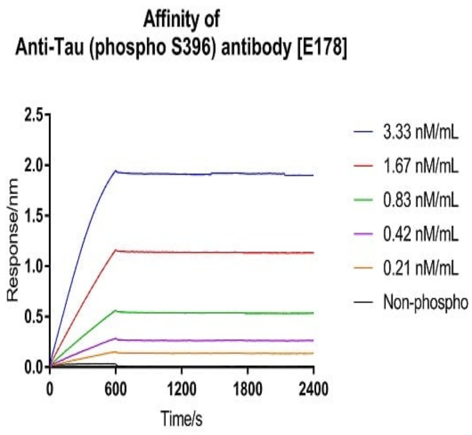

Affinity Purification - Anti-Tau (phospho S396) antibody [E178] (ab32057)

Biotinylated Human Tau (pS396) [0.05 μg/ml] was loaded to SA biosensor on Fortebio RED96e Machine, then associate with recombinant Anti-Tau (phospho S396) antibody [E178] in serial concentration points [3.33, 1.67, 0.83, 0.42, 0.21 nM/mL] by 2-fold dilution, next to dissociate in blank testing buffer [0.1% BSA in PBST (0.05%Tween-20)]. Calculated signals had already subtracted blank control, curve fitting using 1:1 binding model. Non-phospho Tau protein's association and dissociation were also showed in graph. KD(M) value of Anti-Tau (phospho S396) antibody [E178] is 2.08E-11

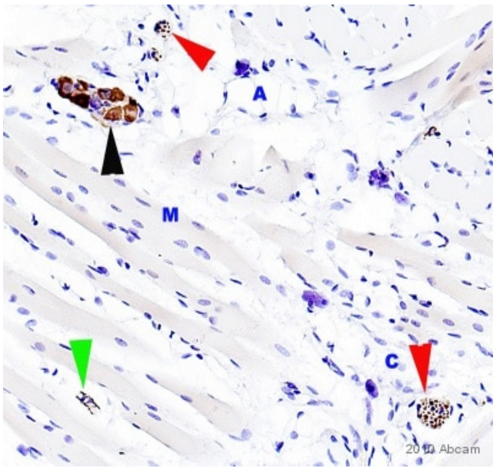

Immunohistochemistry (Formalin/PFA-fixed paraffin-embedded sections) - Anti-Tau (phospho S396) antibody [E178] (ab32057)

This image is courtesy of Carl Hobbs, King's College London, United Kingdom

Immunohistochemistical detection of Tau antibody [E178] (ab32057) on formaldehyde fixed paraffin-embedded rat tongue sectionsAntigen retrieval step: Heat mediated. Buffer Used: Citric acid pH6. Permeabilization: None. Primary antibody incubated at 1/1000 for 2 hours @ 21°C in TBS/BSA/azide. Secondary antibody: anti Rabbit IgG Conjugated to Biotin (1/200). A strong pattern of immunostaining which appears to be mostly localised to nerve fibres and their cell bodies (Islet of Langerhans cells are also positive). In submitted image of central area of tongue coloured arrowheads indicate features: red for nerves cut in cross-section (T/S), each brown dot representing a single axon green for what appears to me to be small nerve fibres wrapping around a partial muscle fibre black for a Ganglion containing seven positive nerve cell bodies. Surrounding these are collagen fibres (C), adipocytes (A) and skeletal/striated muscle fibres in L/S (M-

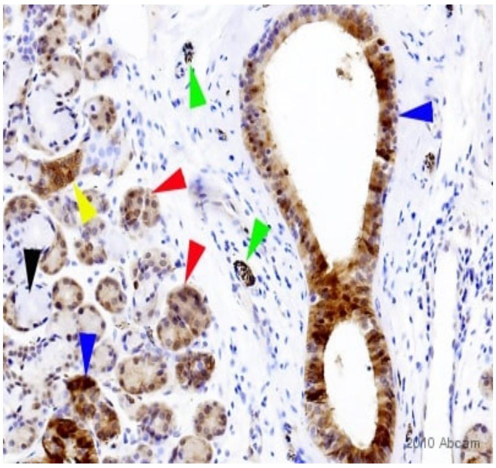

Immunohistochemistry (Formalin/PFA-fixed paraffin-embedded sections) - Anti-Tau (phospho S396) antibody [E178] (ab32057)

This image is courtesy of Carl Hobbs, King's College London, United Kingdom

Immunohistochemistical detection of Tau antibody [E178] (ab32057) on formaldehyde-fixed paraffin-embedded human salivary gland sections. Antigen retrieval step: Heat mediated. Buffer Used: Citric acid pH6. Permeabilization: No. Blocking step: 1% BSA for 10 mins @ 21°C. ab32057 incubated at 1/1000 for 2 hours @ 21°C in TBS/BSA/azide. Secondary antibody: anti rabbit IgG conjugated to Biotin (1/200). NB: An interesting pattern of positivity that seems to be supported by the Human Protein Atlas. Coloured arrowheads in the submitted image indicate features: red for positive serous glands, blue for positive intra-lobular collecting ducts, black for negative mucous glands (there is a serous demilune around this acinus), yellow for intralobular collecting ducts, green for nerve tracks in the interlobular areas, blue for positive interlobular collecting ducts. There appears to be a population of positive nuclei but this may b

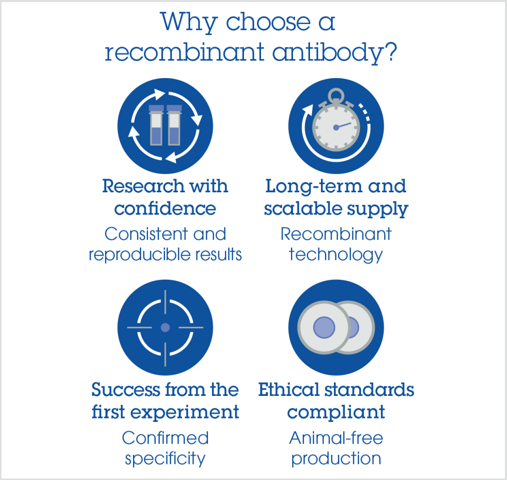

Anti-Tau antibody [E178] (ab32057)

Please note: All products are "FOR RESEARCH USE ONLY. NOT FOR USE IN DIAGNOSTIC PROCEDURES"

Our Abpromise to you: Quality guaranteed and expert technical support

- Replacement or refund for products not performing as stated on the datasheet
- Valid for 12 months from date of delivery
- Response to your inquiry within 24 hours

- We provide support in Chinese, English, French, German, Japanese and Spanish
- Extensive multi-media technical resources to help you
- We investigate all quality concerns to ensure our products perform to the highest standards

If the product does not perform as described on this datasheet, we will offer a refund or replacement. For full details of the Abpromise, please visit **https://www.abcam.com/abpromise** or contact our technical team.

Terms and conditions

- Guarantee only valid for products bought direct from Abcam or one of our authorized distributors
