# Supplementary material for: Neuroprotection and Mechanism of Gas-miR36-5p from Gastrodia elata in an Alzheimer’s Disease Model by Regulating Glycogen Synthase Kinase-3β
Source: Int J Mol Sci. 2023 Dec 9;24(24):17295. doi: 10.3390/ijms242417295 (PMC10744203; doi:10.3390/ijms242417295)
Supplement: Supplementary file 1 [file ijms-24-17295-s001.zip › Datasheets of Tau related primary antibody/Abcam p-Tau Thr 181 254409.pdf]

abcam

Product datasheet

Anti-Tau (phospho T181) antibody [EPR23506-107] ab254409

Recombinant RabMAb

★★★★★ **3 Abreviews** **1 References** 12 Images

Overview

**Product name**	Anti-Tau (phospho T181) antibody [EPR23506-107]
**Description**	Rabbit monoclonal [EPR23506-107] to Tau (phospho T181)
**Host species**	Rabbit
**Specificity**	The specificity of this antibody refers to P10636-8.
**Tested applications**	**Suitable for:** WB, Dot blot, IHC-P, IHC-Fr, IP, Indirect ELISA<br>**Unsuitable for:** ICC/IF
**Species reactivity**	**Reacts with:** Mouse, Rat, Human
**Immunogen**	Synthetic peptide. This information is proprietary to Abcam and/or its suppliers.
**Positive control**	WB: Mouse and rat brain tissue lysate. IHC-P: Mouse and rat cerebrum tissue. IHC-Fr: Mouse and rat cerebrum tissue. IP: Mouse and rat brain tissue lysate.
**General notes**	This product is a recombinant monoclonal antibody, which offers several advantages including:<br>- High batch-to-batch consistency and reproducibility<br>- Improved sensitivity and specificity<br>- Long-term security of supply<br>- Animal-free production<br>For more information **see here**.<br>Our RabMAb® technology is a patented hybridoma-based technology for making rabbit monoclonal antibodies. For details on our patents, please refer to **RabMAb® patents**.

Properties

**Form**	Liquid
**Storage instructions**	Shipped at 4°C. Store at +4°C short term (1-2 weeks). Upon delivery aliquot. Store at -20°C long term. Avoid freeze / thaw cycle.
**Dissociation constant (K_D)**	$K_D = 6.41 \times 10^{-11}$ M

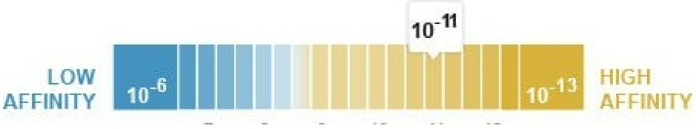

Learn more about K_D

Storage buffer Preservative: 0.01% Sodium azide
Constituents: 59.94% PBS, 40% Glycerol (glycerin, glycerine), 0.05% BSA

Purity Protein A purified

Clonality Monoclonal

Clone number EPR23506-107

Isotype IgG

Applications

The Abpromise guarantee Our **Abpromise guarantee** covers the use of ab254409 in the following tested applications.

The application notes include recommended starting dilutions; optimal dilutions/concentrations should be determined by the end user.

Application	Abreviews	Notes
**WB**	(2)	1/1000. Detects a band of approximately 50-70 kDa (predicted molecular weight: 78 kDa).
**Dot blot**		1/1000.
**IHC-P**	(1)	1/5000. Perform heat mediated antigen retrieval with Tris/EDTA buffer pH 9.0 before commencing with IHC staining protocol.
**IHC-Fr**		1/50.<br>Heat mediated antigen retrieval using sodium citrate buffer (10mM citrate pH 6.0 + 0.05% Tween-20).
**IP**		1/30.
**AP**		Use at an assay dependent concentration.<br>**Antibody concentration range -** 6.67, 3.33, 1.67, 0.83, 0.42, 0 nM/mL
**Indirect ELISA**		Use at an assay dependent concentration. Use at 62.5 ng /mL.

Application notes Is unsuitable for ICC/IF.

Target

Function Promotes microtubule assembly and stability, and might be involved in the establishment and maintenance of neuronal polarity. The C-terminus binds axonal microtubules while the N-terminus binds neural plasma membrane components, suggesting that tau functions as a linker protein between both. Axonal polarity is predetermined by tau localization (in the neuronal cell) in the domain of the cell body defined by the centrosome. The short isoforms allow plasticity of the cytoskeleton whereas the longer isoforms may preferentially play a role in its stabilization.

Tissue specificity Expressed in neurons. Isoform PNS-tau is expressed in the peripheral nervous system while the others are expressed in the central nervous system.

Involvement in disease

Note=In Alzheimer disease, the neuronal cytoskeleton in the brain is progressively disrupted and replaced by tangles of paired helical filaments (PHF) and straight filaments, mainly composed of hyperphosphorylated forms of TAU (PHF-TAU or AD P-TAU).
Defects in MAPT are a cause of frontotemporal dementia (FTD) [MIM:600274]; also called frontotemporal dementia (FTD), pallido-ponto-nigral degeneration (PPND) or historically termed Pick complex. This form of frontotemporal dementia is characterized by presenile dementia with behavioral changes, deterioration of cognitive capacities and loss of memory. In some cases, parkinsonian symptoms are prominent. Neuropathological changes include frontotemporal atrophy often associated with atrophy of the basal ganglia, substantia nigra, amygdala. In most cases, protein tau deposits are found in glial cells and/or neurons.
Defects in MAPT are a cause of Pick disease of the brain (PIDB) [MIM:172700]. It is a rare form of dementia pathologically defined by severe atrophy, neuronal loss and gliosis. It is characterized by the occurrence of tau-positive inclusions, swollen neurons (Pick cells) and argentophilic neuronal inclusions known as Pick bodies that disproportionally affect the frontal and temporal cortical regions. Clinical features include aphasia, apraxia, confusion, anomia, memory loss and personality deterioration.
Note=Defects in MAPT are a cause of corticobasal degeneration (CBD). It is marked by extrapyramidal signs and apraxia and can be associated with memory loss. Neuropathologic features may overlap Alzheimer disease, progressive supranuclear palsy, and Parkinson disease.
Defects in MAPT are a cause of progressive supranuclear palsy type 1 (PSNP1) [MIM:601104, 260540]; also abbreviated as PSP and also known as Steele-Richardson-Olszewski syndrome. PSNP1 is characterized by akinetic-rigid syndrome, supranuclear gaze palsy, pyramidal tract dysfunction, pseudobulbar signs and cognitive capacities deterioration. Neurofibrillary tangles and gliosis but no amyloid plaques are found in diseased brains. Most cases appear to be sporadic, with a significant association with a common haplotype including the MAPT gene and the flanking regions. Familial cases show an autosomal dominant pattern of transmission with incomplete penetrance; genetic analysis of a few cases showed the occurrence of tau mutations, including a deletion of Asn-613.

Sequence similarities

Contains 4 Tau/MAP repeats.

Developmental stage

Four-repeat (type II) tau is expressed in an adult-specific manner and is not found in fetal brain, whereas three-repeat (type I) tau is found in both adult and fetal brain.

Domain

The tau/MAP repeat binds to tubulin. Type I isoforms contain 3 repeats while type II isoforms contain 4 repeats.

Post-translational modifications

Phosphorylation at serine and threonine residues in S-P or T-P motifs by proline-directed protein kinases (PDPK: CDK1, CDK5, GSK-3, MAPK) (only 2-3 sites per protein in interphase, seven-fold increase in mitosis, and in PHF-tau), and at serine residues in K-X-G-S motifs by MAP/microtubule affinity-regulating kinase (MARK) in Alzheimer diseased brains. Phosphorylation decreases with age. Phosphorylation within tau's repeat domain or in flanking regions seems to reduce tau's interaction with, respectively, microtubules or plasma membrane components. Phosphorylation on Ser-610, Ser-622, Ser-641 and Ser-673 in several isoforms during mitosis.
Polyubiquitinated. Requires functional TRAF6 and may provoke SQSTM1-dependent degradation by the proteasome (By similarity). PHF-tau can be modified by three different forms of polyubiquitination. 'Lys-48'-linked polyubiquitination is the major form, 'Lys-6'-linked and 'Lys-11'-linked polyubiquitination also occur.
Glycation of PHF-tau, but not normal brain tau. Glycation is a non-enzymatic post-translational modification that involves a covalent linkage between a sugar and an amino group of a protein molecule forming ketoamine. Subsequent oxidation, fragmentation and/or cross-linking of ketoamine leads to the production of advanced glycation endproducts (AGES). Glycation may

play a role in stabilizing PHF aggregation leading to tangle formation in AD.

Cellular localization Cytoplasm > cytosol. Cell membrane. Cytoplasm > cytoskeleton. Cell projection > axon. Mostly found in the axons of neurons, in the cytosol and in association with plasma membrane components.

Form There are 9 isoforms produced by alternative splicing.

Images

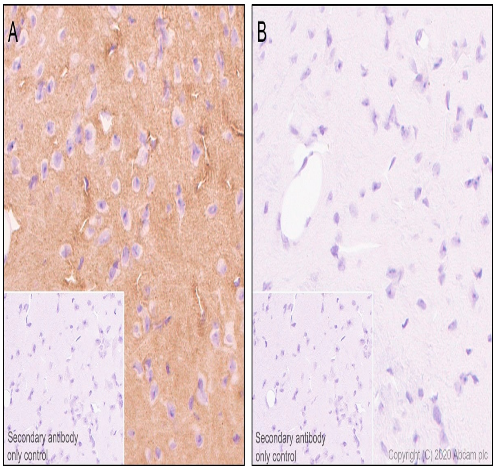

Immunohistochemistry (Formalin/PFA-fixed paraffin-embedded sections) - Anti-Tau (phospho T181) antibody [EPR23506-107] (ab254409)

Immunohistochemical analysis of paraffin-embedded mouse cerebrum tissue labeling Tau (phospho T181) with ab254409 at 1/5000 dilution, followed by Rabbit specific IHC polymer detection kit HRP/DAB (**ab209101**). Positive staining on mouse cerebrum without alkaline phosphatase treatment (image A). No signal was detected when tissues were treated with alkaline phosphatase (image B). Counter stained with hematoxylin.

Secondary antibody only control: Used PBS instead of primary antibody, secondary antibody is Rabbit specific IHC polymer detection kit HRP/DAB (**ab209101**).

Heat mediated antigen retrieval using **ab93684** (Tris/EDTA buffer, pH 9.0).

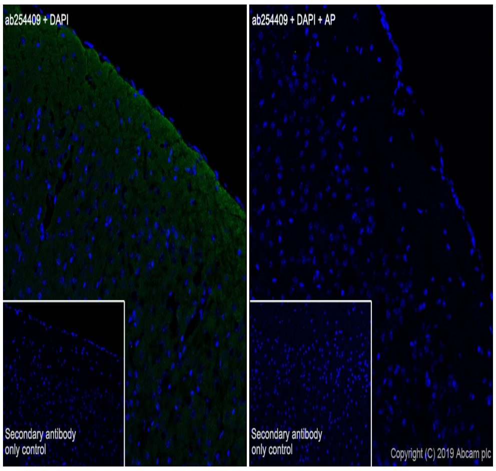

Immunohistochemistry (Frozen sections) - Anti-Tau (phospho T181) antibody [EPR23506-107] (ab254409)

Immunohistochemical analysis of frozen section of 4% PFA-fixed, 0.2% Triton X-100 permeabilized mouse cerebrum tissue labeling Tau (phospho T181) with ab254409 at 1/50 dilution, followed by **ab150077** Goat Anti-Rabbit IgG H&L (Alexa Fluor® 488) at 1/1000 dilution (Green). Positive staining on mouse cerebrum without alkaline phosphatase treatment (image A). No signal was detected when tissues were treated with alkaline phosphatase (image B). The nuclear counterstain is DAPI (Blue).

Secondary antibody only control: Used PBS instead of primary antibody, secondary antibody is **ab150077** Goat Anti-Rabbit IgG H&L (Alexa Fluor® 488) at 1/1000 dilution.

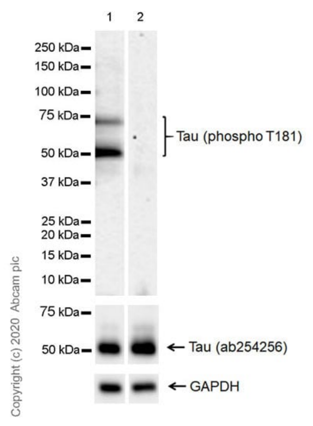

Western blot - Anti-Tau (phospho T181) antibody [EPR23506-107] (ab254409)

All lanes : Anti-Tau (phospho T181) antibody [EPR23506-107] (ab254409) at 1/1000 dilution

Lane 1 : Mouse brain tissue lysate
Lane 2 : Mouse brain tissue lysate (phosphatase treated membrane)

Lysates/proteins at 20 µg per lane.

Secondary
All lanes : Goat Anti-Rabbit IgG, (H+L), Peroxidase conjugated (**ab97051**) at 1/100000 dilution

Predicted band size: 78 kDa
Observed band size: 50-70 kDa

Blocking and dilution buffer: 5% NFDM/TBST.

Exposure time: 3 minutes.

The molecular weight observed is consistent with what has been described in the literature (PMID: 21437732).

This blot was developed using a higher sensitivity ECL substrate.

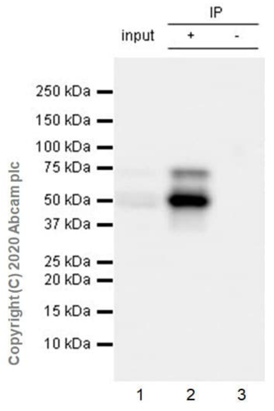

Immunoprecipitation - Anti-Tau (phospho T181) antibody [EPR23506-107] (ab254409)

Tau (phospho T181) was immunoprecipitated from 0.35 mg of mouse brain tissue lysate with ab254409 at 1/30 dilution. Western blot was performed from the immunoprecipitate using ab254409 at 1/1000 dilution. VeriBlot for IP Detection Reagent (HRP) (**ab131366**), was used as secondary antibody at 1/5000 dilution.

Lane 1: Mouse brain tissue lysate 10µg (Input).
Lane 2: ab254409 IP in mouse brain tissue lysate.
Lane 3: Rabbit monoclonal IgG (**ab172730**) instead of ab254409 in mouse brain tissue lysate.

Blocking and dilution buffer and concentration: 5% NFDM/TBST.
Exposure time: 5.5 seconds.

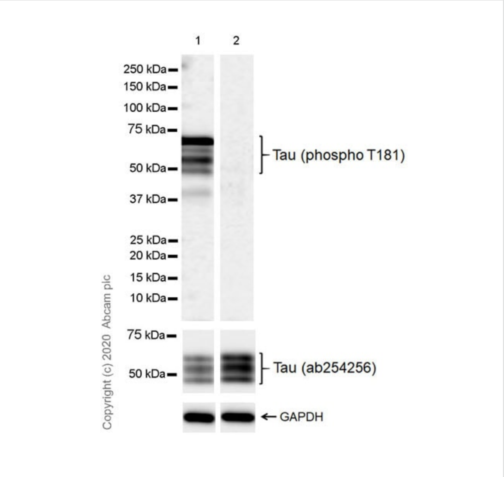

Western blot - Anti-Tau (phospho T181) antibody [EPR23506-107] (ab254409)

All lanes : Anti-Tau (phospho T181) antibody [EPR23506-107] (ab254409) at 1/1000 dilution

Lane 1 : Rat brain tissue lysate
Lane 2 : Rat brain tissue lysate (phosphatase treated membrane)

Lysates/proteins at 20 µg per lane.

Secondary
All lanes : Goat Anti-Rabbit IgG, (H+L), Peroxidase conjugated (**ab97051**) at 1/100000 dilution

Predicted band size: 78 kDa
Observed band size: 50-70 kDa

Blocking and dilution buffer: 5% NFDM/TBST.

Exposure time: 3 minutes.

The molecular weight observed is consistent with what has been described in the literature (PMID: 21437732).

This blot was developed using a higher sensitivity ECL substrate.

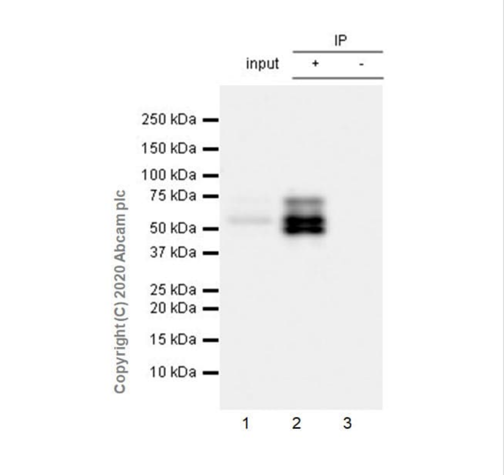

Immunoprecipitation - Anti-Tau (phospho T181) antibody [EPR23506-107] (ab254409)

Tau (phospho T181) was immunoprecipitated from 0.35 mg of rat brain tissue lysate with ab254409 at 1/30 dilution. Western blot was performed from the immunoprecipitate using ab254409 at 1/1000 dilution. VeriBlot for IP Detection Reagent (HRP)(**ab131366**), was used as secondary antibody at 1/5000 dilution.

Lane 1: Rat brain tissue lysate 10µg (Input).
Lane 2: ab254409 IP in rat brain tissue lysate.
Lane 3: Rabbit monoclonal IgG (**ab172730**) instead of ab254409 in rat brain tissue lysate.

Blocking and dilution buffer and concentration: 5% NFDM/TBST.
Exposure time: 10 seconds.

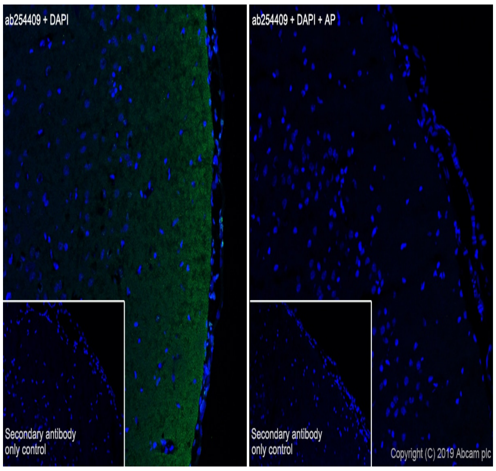

Immunohistochemistry (Frozen sections) - Anti-Tau (phospho T181) antibody [EPR23506-107] (ab254409)

Immunohistochemical analysis of frozen section of 4% PFA-fixed, 0.2% Triton X-100 permeabilized rat cerebrum tissue labeling Tau (phospho T181) with ab254409 at 1/50 dilution, followed by **ab150077** Goat Anti-Rabbit IgG H&L (Alexa Fluor® 488) at 1/1000 dilution (Green). Positive staining on rat cerebrum without alkaline phosphatase treatment (image A). Nearly no signal was detected when tissues were treated with alkaline phosphatase (image B). The nuclear counterstain is DAPI (Blue).

Secondary antibody only control: Used PBS instead of primary antibody, secondary antibody is **ab150077** Goat Anti-Rabbit IgG H&L (Alexa Fluor® 488) at 1/1000 dilution.

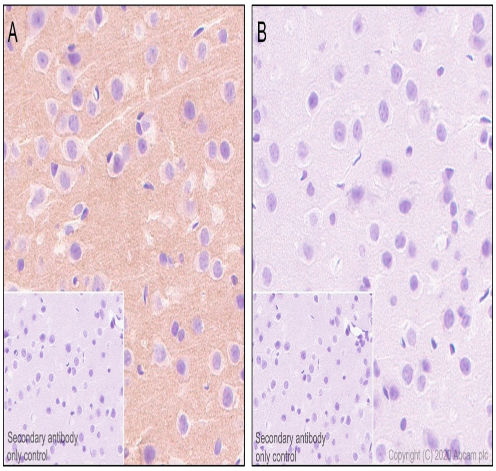

Immunohistochemistry (Formalin/PFA-fixed paraffin-embedded sections) - Anti-Tau (phospho T181) antibody [EPR23506-107] (ab254409)

Immunohistochemical analysis of paraffin-embedded rat cerebrum tissue labeling Tau (phospho T181) with ab254409 at 1/5000 dilution, followed by Rabbit specific IHC polymer detection kit HRP/DAB (**ab209101**). Positive staining on rat cerebrum without alkaline phosphatase treatment (image A, PMID: 30279741). No signal was detected when tissues were treated with alkaline phosphatase (image B). Counter stained with hematoxylin.

Secondary antibody only control: Used PBS instead of primary antibody, secondary antibody is Rabbit specific IHC polymer detection kit HRP/DAB (**ab209101**).

Heat mediated antigen retrieval using **ab93684** (Tris/EDTA buffer, pH 9.0).

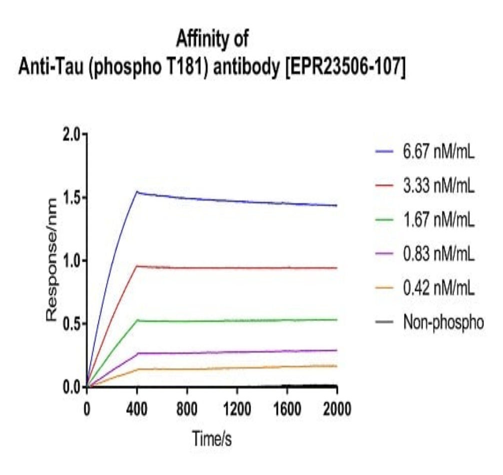

Affinity Purification - Anti-Tau (phospho T181) antibody [EPR23506-107] (ab254409)

Biotinylated Human Tau phospho T181 [0.5 μg/ml] was loaded to SA biosensor on Fortebio RED96e Machine, then associate with recombinant Anti-Tau (phospho T181) antibody [EPR23506-107] in serial concentration points [6.67, 3.33, 1.67, 0.83, 0.42 nM/mL] by 2-fold dilution, next to dissociate in blank testing buffer [0.1% BSA in PBST (0.05%Tween-20)]. Calculated signals had already subtracted blank control, curve fitting using 1:1 binding model. Non-phospho Tau protein's association and dissociation were also showed in graph. KD (M) value of Anti-Tau (phospho T181) antibody [EPR23506-107] is 6.41E-11

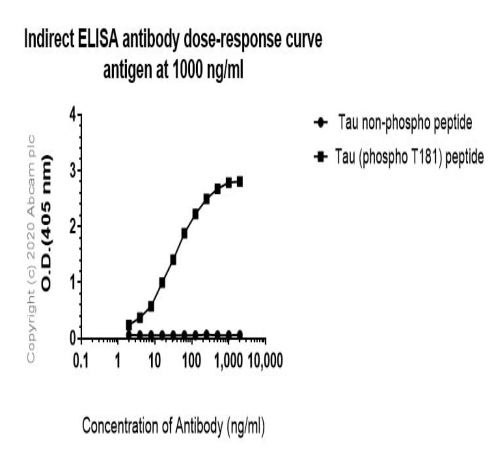

Indirect ELISA - Anti-Tau (phospho T181) antibody [EPR23506-107] (ab254409)

Antigen: Mouse Tau non-phospho peptide□Mouse Tau (phospho T181) peptide.

Antigen concentration: 1000 ng/mL.

ab254409 used at 0 - 2000 ng/mL.

Secondary antibody: Alkaline Phosphatase-conjugated AffiniPure Goat Anti-Rabbit IgG (H+L) at 1/2500 dilution.

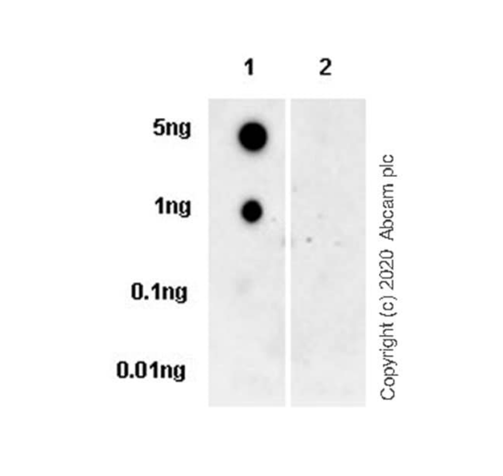

Dot Blot - Anti-Tau (phospho T181) antibody [EPR23506-107] (ab254409)

Dot blot analysis of Tau (phospho T181) labeled with ab254409 at 1/1000 dilution.

Lane 1: Tau (phospho T181) peptide (aa 177-190).

Lane 2: Tau non-phospho peptide (aa 174-190).

Goat Anti-Rabbit IgG H&L (HRP) (**ab97051**) at 1/100000 dilution was used as secondary antibody.

Blocking and dilution buffer: 5% NFDM/TBST.

Exposure time: 3 minutes.

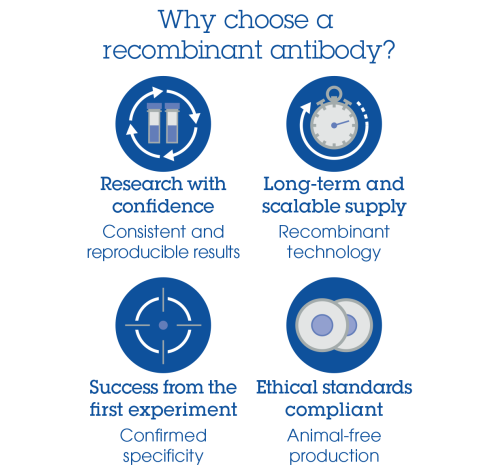

Anti-Tau (phospho T181) antibody [EPR23506-107] (ab254409)

Please note: All products are "FOR RESEARCH USE ONLY. NOT FOR USE IN DIAGNOSTIC PROCEDURES"

Our Abpromise to you: Quality guaranteed and expert technical support

- Replacement or refund for products not performing as stated on the datasheet
- Valid for 12 months from date of delivery
- Response to your inquiry within 24 hours

- We provide support in Chinese, English, French, German, Japanese and Spanish
- Extensive multi-media technical resources to help you
- We investigate all quality concerns to ensure our products perform to the highest standards

If the product does not perform as described on this datasheet, we will offer a refund or replacement. For full details of the Abpromise, please visit **https://www.abcam.com/abpromise** or contact our technical team.

Terms and conditions

- Guarantee only valid for products bought direct from Abcam or one of our authorized distributors
