# Supplementary material for: Neuroprotection and Mechanism of Gas-miR36-5p from Gastrodia elata in an Alzheimer’s Disease Model by Regulating Glycogen Synthase Kinase-3β
Source: Int J Mol Sci. 2023 Dec 9;24(24):17295. doi: 10.3390/ijms242417295 (PMC10744203; doi:10.3390/ijms242417295)
Supplement: Supplementary file 1 [file ijms-24-17295-s001.zip › Datasheets of Tau related primary antibody/Proteintech Tau 66499-1-Ig datasheet.pdf]

For Research Use Only

TAU Monoclonal antibody

Catalog Number:66499-1-Ig Featured Product 11 Publications

proteintech® Antibodies | ELISA kits | Proteins
www.ptglab.com

Basic Information

Catalog Number:
66499-1-Ig

Size:
150ul , Concentration: 1100 μg/ml by Nanodrop and 1000 μg/ml by Bradford method using BSA as the standard;

Source:
Mouse

Isotype:
IgG2c

Immunogen Catalog Number:
AG21926

GenBank Accession Number:
BC000558

GeneID (NCBI):
4137

Full Name:
microtubule-associated protein tau

Calculated MW:
37-46, 79-81 kDa

Observed MW:
100 kDa

Purification Method:
Protein A purification

CloneNo.:
1E9A8

Recommended Dilutions:
WB 1:1000-1:30000
IHC 1:200-1:800

Applications

Tested Applications:
IHC, WB, ELISA

Cited Applications:
IF, WB

Species Specificity:
Human, mouse, rat, pig

Cited Species:
human, rat, mouse

Note-IHC: suggested antigen retrieval with TE buffer pH 9.0; (*) Alternatively, antigen retrieval may be performed with citrate buffer pH 6.0

Positive Controls:

WB : HeLa cells, Y79 cells, pig brain tissue, SH-SY5Y cells, U-251 cells, Neuro-2a cells, rat brain tissue, mouse brain tissue

IHC : human gliomas tissue, mouse brain tissue

Background Information

The microtubule-associated protein TAU (MAPT or TAU) is encoded by MAPT gene, which locates on human chromosome 17q21, binds to the tubulin subunit of microtubule and promotes its assembly and stability. Most TAU is expressed in neurons, and TAU isoform is expressed in the peripheral nervous system while the others are expressed in the central nervous system. TAU links axonal microtubules with C-terminus to neural plasma membrane components with its N-terminus, suggesting the participation in intracellular signal transduction and neuron's development and viability. Various isoforms of Tau exist due to the alternative splicing, and short isoforms around 45-69 kDa and long isoforms around 100-110 kDa have been reported in different literature (PMID:8752131,15965697, 12485403).Present monoclonal anti-Tau antibody can detect approx 100-kDa bands in brain tissues.

Notable Publications

Author	Pubmed ID	Journal	Application
Estibaliz Santiago-Mujika	36606207	J Alzheimers Dis Rep	WB
Jiqu Xu	31050371	J Pineal Res	WB
Nicholas E. Albrecht	35880013	Cell Rep Methods	IF

Storage

Storage:
Store at -20°C. Stable for one year after shipment.
Storage Buffer:
PBS with 0.02% sodium azide and 50% glycerol pH 7.3.
Aliquoting is unnecessary for -20°C storage

***** 20ul sizes contain 0.1% BSA**

Selected Validation Data

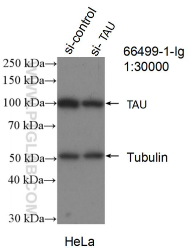

WB result of TAU antibody (66499-1-Ig; 1:30000; incubated at room temperature for 1.5 hours) with sh-Control and sh-TAU transfected HeLa cells.

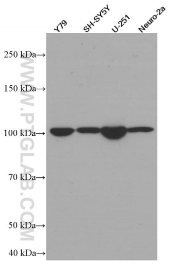

Various lysates were subjected to SDS PAGE followed by western blot with 66499-1-Ig (TAU antibody) at dilution of 1:5000 incubated at room temperature for 1.5 hours.

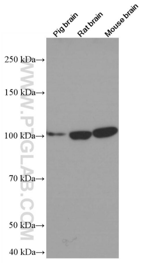

Various lysates were subjected to SDS PAGE followed by western blot with 66499-1-Ig (TAU antibody) at dilution of 1:5000 incubated at room temperature for 1.5 hours.

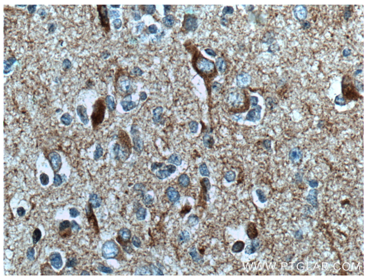

Immunohistochemical analysis of paraffin-embedded human gliomas tissue slide using 66499-1-Ig (TAU antibody) at dilution of 1:400 (under 40x lens. Heat mediated antigen retrieval with Tris-EDTA buffer (pH 9.0).

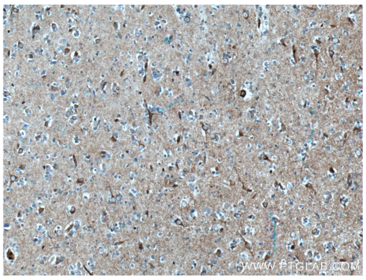

Immunohistochemical analysis of paraffin-embedded human gliomas tissue slide using 66499-1-Ig (TAU antibody) at dilution of 1:400 (under 10x lens. Heat mediated antigen retrieval with Tris-EDTA buffer (pH 9.0).
